# Symmetrical Bispyridinium Compounds Act as Open Channel Blockers of Cation-Selective Ion Channels

**DOI:** 10.1021/acsptsci.3c00308

**Published:** 2024-02-15

**Authors:** Yves Haufe, Dominik Loser, Timm Danker, Annette Nicke

**Affiliations:** † Walther Straub Institute of Pharmacology and Toxicology, Faculty of Medicine, LMU Munich, 80336 Munich, Germany; ‡ NMI Natural and Medical Sciences Institute at the University of Tübingen, 72770 Reutlingen, Germany

**Keywords:** nicotinic acetylcholine receptor, organophosphate poisoning, Xenopus laevis oocytes, bispyridinium, open channel block, allosteric modulator

## Abstract

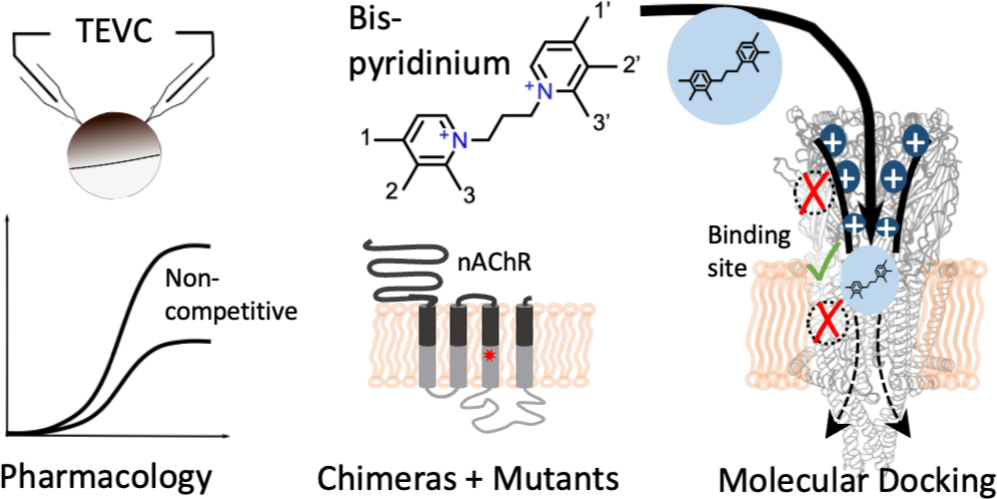

Current treatments against organophosphate poisoning (OPP) do not directly address effects mediated by the overstimulation of nicotinic acetylcholine receptors (nAChR). Non-oxime bispyridinium compounds (BPC) promote acetylcholine esterase-independent recovery of organophosphate-induced paralysis. Here, we test the hypothesis that they act by positive modulatory action on nAChRs. Using two-electrode voltage clamp analysis in combination with mutagenesis and molecular docking analysis, the potency and molecular mode of action of a series of nine BPCs was investigated on human α7 and muscle-type nAChRs expressed in *Xenopus laevis* oocytes. The investigated BPCs inhibited α7 and/or muscle-type nAChRs with IC_50_ values in the high nanomolar to high micromolar range. Further analysis of the most potent analogues revealed a noncompetitive, voltage-dependent inhibition. Co-application with the α7-selective positive allosteric modulator PNU120596 and generation of α7/5HT3 receptor chimeras excluded direct interaction with the PNU120596 binding site and binding to the extracellular domain of the α7 nAChR, suggesting that they act as open channel blockers (OCBs). Molecular docking supported by mutagenesis localized the BPC binding area in the outer channel vestibule between the extracellular and transmembrane domains. Analysis of BPC action on other cation-selective channels suggests a rather nonspecific inhibition of pentameric cation channels. BPCs have been shown to ameliorate organophosphate-induced paralysis *in vitro* and *in vivo*. Our data support molecular action as OCBs at α7 and muscle-type nAChRs and suggest that their positive physiological effects are more complex than anticipated and require further investigation.

Organophosphate poisoning (OPP) by pesticide exposure or by nerve agents represents a serious condition with insufficient treatment options and possible long-term impairment.^1−3^ The primary effect of organophosphate compounds (OPCs) is an irreversible block of the pivotal enzyme acetylcholine esterase (AChE), resulting in the accumulation of acetylcholine and overstimulation of both nicotinic (nAChR) and muscarinic (mAChR) acetylcholine receptors in the CNS and PNS (cholinergic syndrome). Life-threatening effects include bronchorrhea, bradycardia, inhibition of respiration, and muscle paralysis and require rapid treatment.^4^ The standard therapy comprises the mAChR antagonist atropine, AChE reactivating pyridinium oximes, and symptomatic treatments such as benzodiazepines and ventilation.^3^ However, the use of oximes is limited by the fast formation of covalently linked OP-AChE complexes (so-called “aging”), and the efficiency of the available oxims critically depends on the respective OPC.^5^ Treatment regimes showed only limited improvements in the last decades. nAChR-mediated effects at the neuromuscular junction represent a major therapeutic gap as nAChR overstimulation and desensitization lead eventually to a depolarization block of voltage-gated sodium channels.^6^ The resulting muscle paralysis might require ventilation for weeks, and respiratory failure is the most common cause of death.^4^ Besides these prevalent muscle effects, acute and chronic exposure to OPCs is associated with polyneuropathy, neurodegeneration, and memory impairment, suggesting a possible involvement of neuronal nAChRs.^7−9^ Beneficial effects of non-oxime bispyridinium compounds (BPCs) have been reported in *ex vivo* experiments with mammalian muscle preparations^6,10,11^ and in animal experiments.^10,12^ However, different modes of action are proposed in the literature. Based on single channel analyses on muscle nAChRs, early studies indicated an action as open channel blocker (OCB)^6,10,13^ that prevents receptor activation and desensitization. Later studies found competitive binding at the orthosteric ACh-binding at nAChRs from *Torpedo californica,*(14) and the involvement of a stabilizing, allosteric binding site was proposed for BPCs with longer linkers.^15^ An allosteric modulation by selected BPCs via a binding site in the extracellular domain (ECD) and competitive binding was recently supported by computational approaches.^16^ Studies on a α7 nAChR-expressing CHO cell line showed that BPCs act as positive allosteric modulators (PAMs) and promote nAChR resensitization.^17^ However, whether a similar mechanism is possible for muscle-type nAChR is not clear.

nAChRs belong to the cys-loop receptor superfamily of pentameric ligand-gated ion channels (pLGICs), which also includes cationic serotonin (5HT3) and anionic glycine and γ-amino butyric acid neurotransmitter (GABA) receptors. While PAMs of GABA receptors such as barbiturates and benzodiazepines represent important drugs, this concept has been less exploited for other pLGICs.

The best studied nAChR subtype in regard to allosteric modulation is the neuronal α7 nAChR and most of the PAMs described for nAChRs act exclusively on this receptor.^18^ Type I PAMs potentiate agonist responses without changing receptor kinetics, while type II PAMs, like PNU120596, additionally change the equilibrium between open and desensitized receptor states and thereby can prevent desensitization and/or promote resensitization in the presence of an orthosteric agonist.^19^ Identification of PAMs that modulate or resensitize muscle-type nAChRs represents an intriguing approach to counteracting OPP.

In this study, we therefore set out to characterize and compare the effects, pharmacological mode of action, and binding sites of selected symmetrical C3-linker BPCs at the human α7 and muscle-type nAChRs.

## Results

### Symmetrical Bispyridinium Compounds Block nAChR Currents with Micromolar Potency

**Figure 1:**
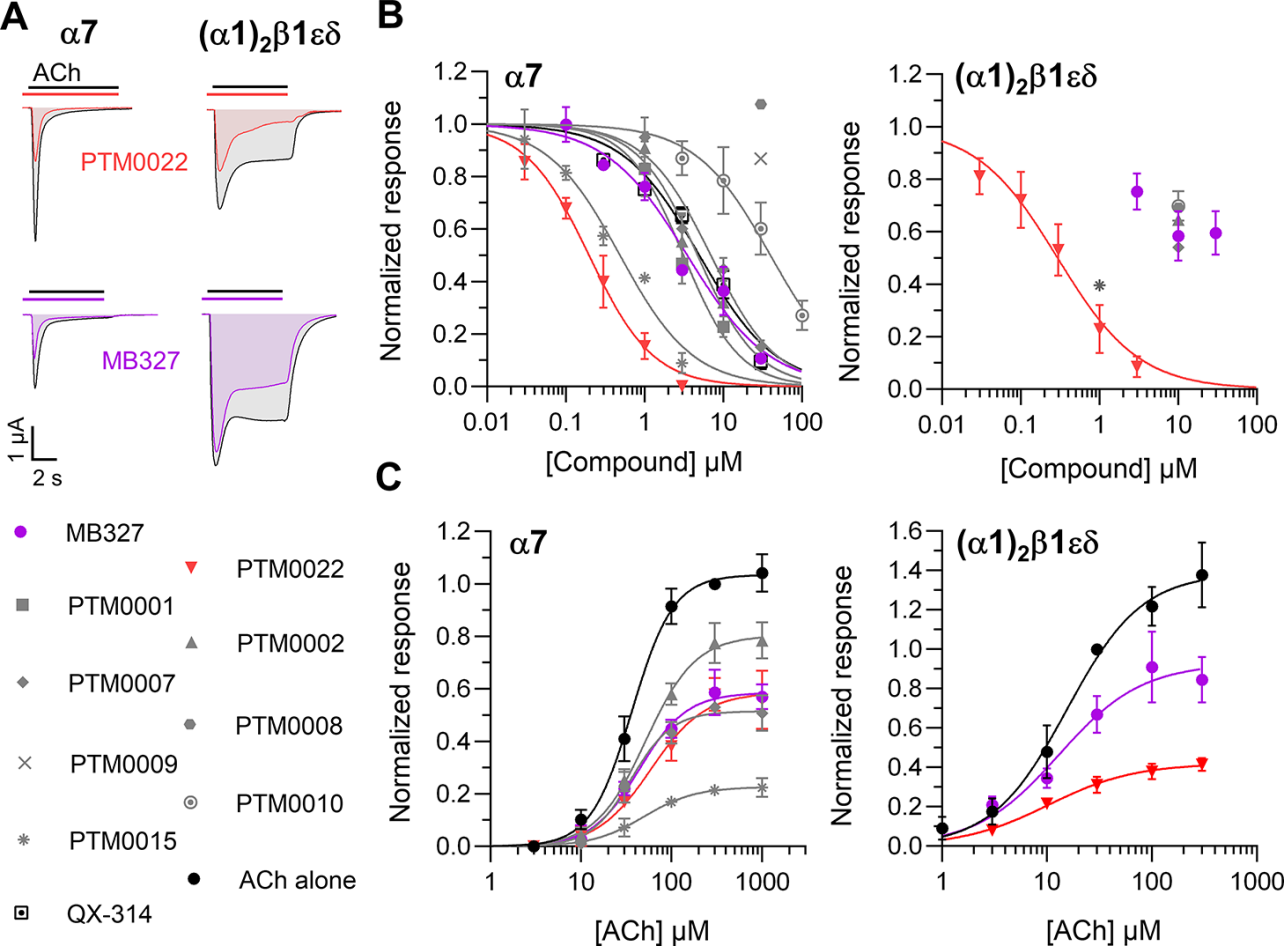
Symmetrical BPCs with a C3 linker inhibit α7 and muscle-type nAChR currents with micromolar potency and in a noncompetitive way. Human α7 and (α1)2β1εδ nAChRs were expressed in *Xenopus laevis* oocytes. Oocytes were clamped at −70 mV, and BPCs were preincubated (20 s) and coapplied with ACh. (A) Representative current traces in response to 100 μM ACh (α7) or 30 μM ACh (muscle-type) before and after application of 300 nM PTM0022 (red) or 3 μM MB327 (magenta). (B) Full dose inhibition curves of BPCs with at least a low micromolar potency at α7 and muscle nAChR subtypes. Error bars represent the SD of the mean from *n* = 3–6 individual oocytes. (C) ACh dose response curves without black filled circles and in the presence of the following concentrations of BPCs: 3 μM MB327, 3 μM PTM0002, 10 μM PTM0007, 3 μM PTM0015, and 300 nM PTM0022. Individual BPCs are indicated in the legend. MB327 and the most potent derivate PTM0022 are colored. Error bars represent the SD from the mean of 5–7 individual oocytes. Best-fit values from parts B and C are shown in Tables 1 and 2.

The symmetrical BPC MB327 has previously been shown to reactivate muscle contractility in an organ model of OPP^6,10,11^ and to act as PAM on the α7 nAChR.^20^ It therefore served as a lead structure for the synthesis of derivatives with different substituents at the aromatic rings,^21^ of which eight compounds (for structures, see Figure 7) were investigated in this study. To directly compare their action on α7 and muscle (α1)_2_β1εδ nAChR subtypes, we expressed both subtypes in *Xenopus laevis* oocytes and determined the BPC effects on ACh-activated currents by two-electrode voltage clamp (TEVC, Figure 1). Unexpectedly, none of the compounds showed a PAM-like action but either blocked both receptors or did not show an effect at the tested concentrations (up to 30 μM). Dose-inhibition analyses at the α7 nAChR revealed IC_50_ values in the high nanomolar to micromolar range for all compounds, except for PTM0008 and PTM0009, which had no effect (Figure 1B and Table 1). While the lead compound MB327 showed intermediate potency (IC_50_ value: 3.5 μM), PTM0022 and PTM0015, which carried two substituents per ring, had up to 17-fold higher potency with IC_50_ values of 0.20 and 0.49 μM, respectively. In agreement with binding studies at the *Torpedo* nAChR,^21^ these two compounds also showed the highest potency at the muscle nAChR, with IC_50_ values just below and around 1 μM, respectively. The estimated IC_50_ values of all other BPCs, including MB327, were above 10 μM and therefore were not determined. To test whether they act as competitive antagonists at the orthosteric ACh binding site, we next compared ACh dose–response curves without and in the presence of BPCs (Figure 1C). These analyses revealed an insurmountable BPC-induced block at both nAChR subtypes, indicating a noncompetitive binding mode. The lead compound MB327 and the most potent compound PTM0022 were selected for further analysis at α7 nAChR.

### BPCs Do Not Interact Directly with the Allosteric PNU120596 Binding Site of the α7 nAChR

**Figure 2:**
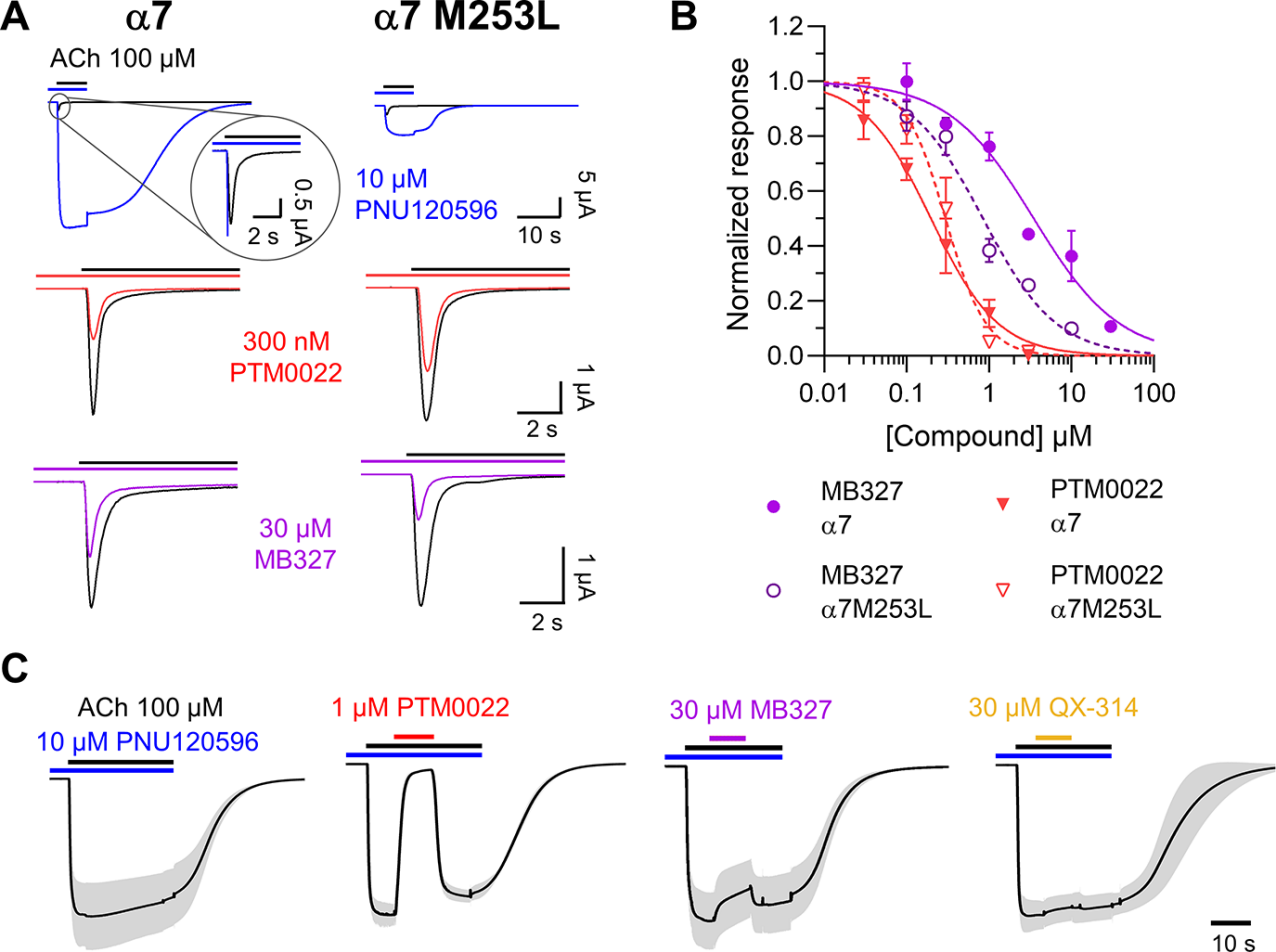
Influence of the α7M253L allosteric binding site mutation on BPC potency and interactions between BPC and PNU120596 binding. (A) Human α7 and α7M253L nAChRs were expressed in *Xenopus laevis* oocytes and clamped at −70 mV. Representative current traces in response to 100 μM ACh (black bar) are shown before (black) and after preincubation (20 s) and coapplication of the indicated compounds (colored lines and bars). Note the more than 10-fold increase of ACh-elicited current amplitude by PNU120596. (B) Dose-inhibition curves for the indicated BPCs at the human α7 and α7 M253L mutant (*n* = 3–6, the mean and SD are shown). Dotted/solid lines represent data from Figure 1B, for comparison. (C) Co-application of BPCs or QX-314 upon sequential preapplication of 10 μM PNU120596 and 100 μM ACh. Averaged current traces from at least five different oocytes are shown. SD is shown in light gray. Current traces were normalized to the ACh-elicited signal before the co-application. PNU125096-amplified signals were between 20 and 30 μA. Note that, due to the normalization, absolute current values are not shown.

MB327 and some of its derivates caused similar positive allosteric effects as the widely used PAM PNU120596 at CHO cells, that stably expressed α7 receptor.^20^ Since minor changes in the receptor, such as the single exchange of an amino acid^22^ or lipid composition,^23,24^ can alter the effect of allosteric modulators dramatically, we next investigated if the inhibitory effects observed in this study could be caused by a negative allosteric mechanism via the well characterized binding site of PNU120596.^25,26^ To this aim, we generated the α7 PAM binding site mutant M253L and tested whether it also affected BPC binding (Figure 2). As shown before, PNU120596 strongly delays α7 desensitization and increases the current amplitude,^27^ while the M253L point mutation reduces the potentiating effect of PNU120596 (as determined by the net current) more than 11-fold^25^ (Figure 2A). Interestingly, the antagonistic potency of MB327 is 4-fold increased on this mutation, whereas the dose inhibition curve of PTM0022 has only a slightly steeper hill coefficient (Figure 2B and Table 3). This suggests that the mutation has a selective effect on MB327 binding and/or its allosteric efficiency. To further test the possibility of a shared allosteric binding site with PNU120596, we investigated the binding kinetics of the selected BPCs by co-application with PNU120596, making use of the prolonged open state of the α7 (Figure 2C). As a control, we used QX-314, a lidocaine derivative that is one of the best-characterized OCBs of some nAChRs.^28,29^ All compounds were applied in concentrations that produced 70–90% block at α7 in the absence of PNU120596. Surprisingly, PTM0022 showed a fast and almost complete block of the PNU120596-potentiated α7 current and a fast unbinding and recovery of the PNU-potentiated current upon washout. Likewise, MB327 showed quickly reversible inhibition, although to a much smaller extent. Unexpectedly, the OCB QX-314 was unable to block at all. The fast effect of PTM0022 compared to the slow washout of PNU120596 upon its removal argues against a direct interaction of both substances at the allosteric binding site, and we therefore tested whether the BPCs act as channel blockers.

**Table 1:** IC_50_ Values and Hill Coefficients (*n*_H_) of BPCs at Human α7 and (α1)_2_β1εδ nAChRs^a^

compound	α7				(α1)_2_β1εδ		
	IC_50_	95% CI μM^a^	IC_50_ ratio	*n*_H_	IC_50_	95% CI μM^a^	*n*_H_
MB327	3.50	2.73–4.51	1.00	–0.84	∼30		
PTM0001	3.13	2.39–4.20	1.10	–1.23			
PTM0002	4.72	3.60–6.27	0.74	–1.14	>10		
PTM0007	6.96	5.44–8.90	0.50	–1.04	>10		
PTM0008	>30				>10		
PTM0009	>30						
PTM0010	39.83	30.95–51.75	0.09	–0.91	>10		
PTM0015	0.49	0.39–0.62	7.14	–0.94	<1*		
PTM0022	0.20	0.17–0.23	17.50	–1.07	0.29	0.23–0.38	–0.84
QX-314	4.98	4.03–6.10	0.70	–0.89			

a Values represent 95% confidence intervals. The corresponding dose–response curves are shown in Figure 1B. IC_50_ ratios relative to MB327 are shown. *Due to limited material, no full DRC was obtained. MB327 and PTM0022 were selected for further in-depth analysis.

**Table 2:** EC_50_ Values and Hill Coefficients (*n*_H_) of ACh in the Presence of the Indicated Concentrations of BPCs at Human α7 and (α1)2β1εδ nAChRs

compound	α7			(α1)_2_β1εδ		
	EC_50_	95% CI μM^a^	*n*_H_	EC_50_	95% CI μM^a^	*n*_H_
ACh	36.5	33.5–40.0	1.90	15.4	13.1–18.8	1.22
+3 μM MB327	43.8	33.6–58.2	1.66			
+10 μM MB327				13.0	8.1–26.1	1.10
+3 μM PTM0002	51.6	42.5–63.2	1.57			
+10 μM PTM0007	35.8	29.2–45.7	1.93			
+3 μM PTM0015	49.2	31.7–82.2	1.53			
+0.3 μM PTM0022	58.8	44.0–81.4	1.46			
+1 μM PTM0022				10.3	7.3–17.2	1.06

a Values represent 95% confidence intervals. The corresponding dose–response curves are shown in Figure 1C.

**Table 3:** IC_50_ Values and Hill Coefficients (*n*_H_) for MB327 and PTM0022 at Human α7 and α7M253L nAChRs

	α7			α7 M253L			
compound	IC_50_	95% CI μM^a^	*n*_H_	IC_50_	95% CI μM^a^	*n*_H_	IC_50_(α7)/IC_50_(M253L)
MB327	3.50	2.73–4.51	–0.84	0.84	0.69–1.03	–0.98	4.17
PTM0022	0.20	0.17–0.23	–1.07	0.30	0.25–0.35	–1.76	0.67

a Values represent 95% confidence intervals. Corresponding dose–inhibition curves are shown in Figure 2B.

### BPCs Act as Channel Blockers

**Figure 3:**
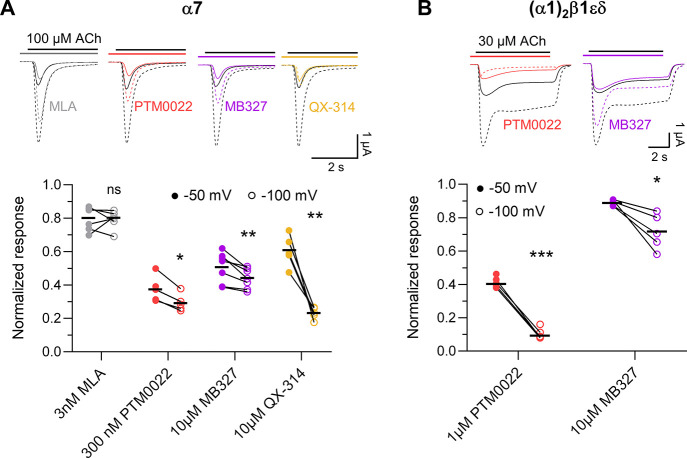
Voltage-dependency of α7 and (α1)_2_β1εδ nAChR inhibition by selected BPCs. Representative current traces and statistical analysis of ACh (100 μM)-induced current responses from α7 (A) and (α1)_2_β1εδ nAChRs (B) before and after inhibition by the indicated compounds. Gray current traces represent equilibrated control currents at −50 mV (solid) and −100 mV (dotted). Colored lines represent the respective responses following 20 s of preincubation and co-application of the indicated compounds with ACh. Note that current traces are larger at −100 mV. Paired analysis comparing normalized responses upon antagonist application at a holding potential of −50 mV (filled symbols) and −100 mV (empty symbols) from 4–6 individual oocytes is shown. Responses were normalized to the currents in the absence of antagonists. Single values are shown, with the mean displayed as a black line. Statistical significance was determined with a paired *t*-test with *p* < 0.05 *, *p* < 0.01 **, and *p* < 0.001 ***. MLA (gray), PTM0022 (red), MB327 (magenta), and QX-314 (gold).

Channel blockers are noncompetitive antagonists that prevent ion flux by physically occluding the channel pore. OCBs are characterized by their use-dependency, meaning that their effect increases with prolonged opening until their binding equilibrium is reached. Channel blockers generally show voltage-dependent inhibition and most cation channel blockers carry a positive charge.^30,31^ Since BPCs are permanently positively charged, we next tested the voltage-dependency of their inhibition by comparing their potency at α7 nAChR-expressing oocytes clamped at −50 and −100 mV (Figure 3). Methyllycaconitine (MLA, 3 nM), an uncharged α7-selective competitive antagonist, was used as a negative control and, as expected, did not show an altered response (Figure 3A). In contrast, the positive control QX-314 showed a significantly stronger inhibition of ACh-evoked responses at −100 mV. Likewise, MB327 and PTM0022 showed a significantly stronger inhibition of α7 responses, and this voltage-dependent inhibition was even more pronounced at the muscle-type nAChR. These data support a channel block as the mode of action of BPCs. Note that the α7 nAChR does not show currents if clamped at positive voltages,^32^ hence, these could not be tested.

### Ion Channel Mutants and Chimeras Support BPC Binding within the Transmembrane Domain

**Figure 4:**
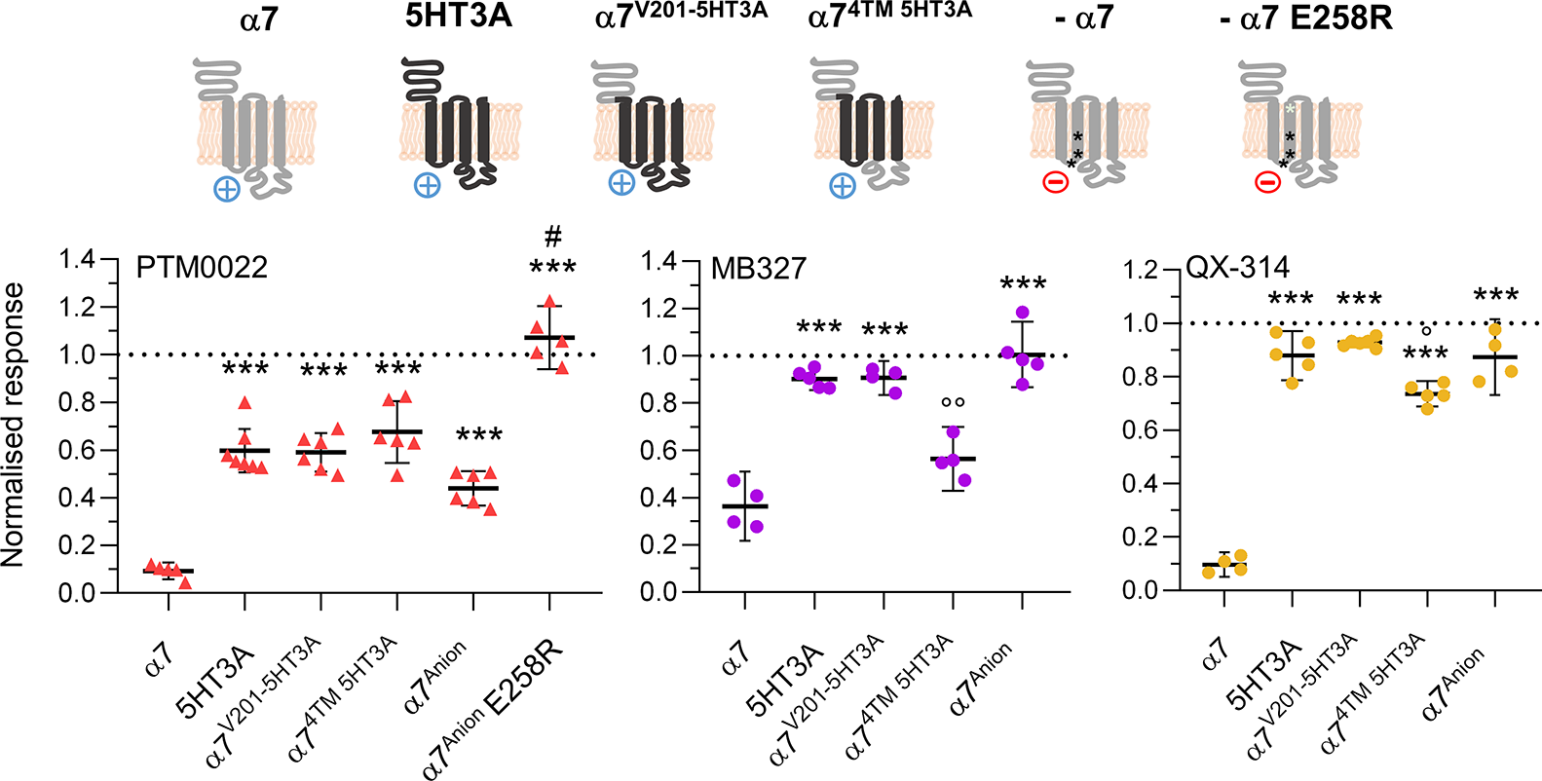
Inhibition of α7 chimeras and mutants by BPCs. Normalized responses of 1 μM PTM0022 (red triangle), 10 μM MB327 (magenta dot), and 30 μM QX-314 (golden dot) at the indicated chimeras and mutants (shown as pictograms, see Table S1 for more details). Single values are shown with the mean displayed as black line. Error bars represent 95% CIs (*n* = 4–6). Statistical analysis was done using Brown–Forsythe and Welch ANOVA with posthoc Dunnett T3 test compared to α7 with *p* < 0.05 *, *p* < 0.01 **, and *p* < 0.001 ***, to −α7 with *p* < 0.001 #, and to 5HT3A with *p* < 0.05°, *p* < 0.01°°. EC_80_ concentration of agonist was applied, and currents were normalized to currents evoked before incubation and co-application of the indicated compound.

To further test our hypothesis that BPCs bind within the channel pore, we generated α7/5HT3 receptor chimeras. Chimeras between these pentameric cation receptors have previously been generated, and essential gating mechanisms were shown to be preserved.^33^ We first compared the potencies of PTM0022, MB327, and the positive control QX-314 at the α7 nAChR and the 5HT3A serotonin receptors. All compounds produced a significantly stronger block of the α7 nAChR (mean responses of α7 vs 5HT3A, respectively, for 1 μM PTM0022: 9.3% vs 59.7%, 10 μM MB327: 36.3% vs 90.2%, 30 μM QX-314: 9.6% vs 90.0%) (Figure 4). We then systematically exchanged domains of the α7 nAChR with those of the 5HT3A receptor and vice versa (Figure 4 and Table S1) to identify regions involved in BPC binding. At the α7–5HT3A chimera (α7 extracellular domain (ECD), α7^V201–5HT3A^^33^), all compounds showed similar inhibition as on the WT 5HT3A receptor, thus excluding a binding area within the α7 ECD. The additional introduction of the intracellular domain (ICD) of the α7 into the 5HT3A (α7^4TM5HT3A^) likewise did not result in a substantial potency increase of PTM0022 or QX-314. MB327, however, showed significantly increased potency on this chimera, indicating that the ICD can influence the binding of some of the compounds. Additional chimeras did not result in functional receptors (see Table S1).

Inhibition of nAChRs by high concentrations of its positively charged agonist ACh is frequently observed and has been attributed to an OCB due to its cation-conducting properties.^34,35^ To explore the possibility that the cationic BPCs also interact with negative residues in the entrance of the channel pore, we generated a previously characterized α7 triple mutant (P236 insertion, E237A, V251T,^32^ α7^Anion^), which functions as a semi-anion channel and should attract less cations into the pore. Neither QX-314 nor MB327 were able to inhibit this mutant at the tested concentrations. In contrast, inhibition by 1 μM PTM0022 was significantly reduced but not eliminated. Substitution of an additional negatively charged residue of the anionic ring at the upper part of the pore by a positively charged residue (E258R, E20′) completely abolished the inhibition by PTM0022.

### Molecular Docking Identifies a High Affinity of BPCs to Binding Sites in the Upper Part of the α7 Channel Pore

**Figure 5:**
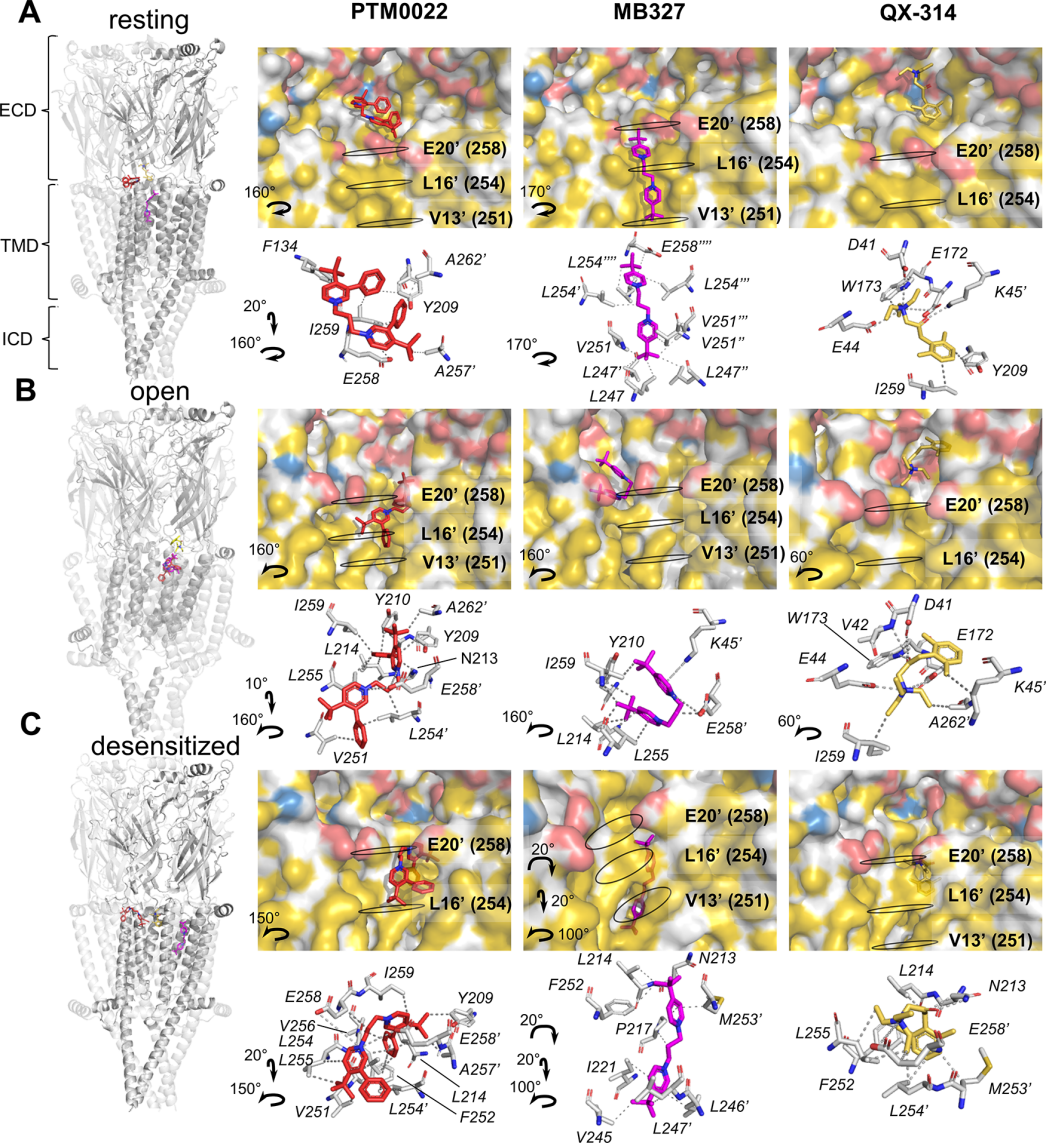
Molecular docking of PTM0022, MB327, and QX-314 in open, closed, and desensitized states of the α7 nAChR. Results with the highest binding energy are shown for PTM0022, MB327, and QX-314 in the resting (PDB ID: 7KOO, A), open (PDB ID: 7KOX, B), and desensitized (PDB ID: 7KOQ, C) states of the α7 nAChR. Cartoon structures of each receptor state with all three compounds are shown on the left. Detailed surface representation and interacting amino acid residues for PTM0022 (red), MB327 (magenta), and OX-314 (yellow) are shown on the right. Color coding follows YRB script (Hagemans et al., 2015) with hydrophobic C atoms in yellow and polar interaction partners in blue (positive) and red (negative). Black rings indicate the position of the labeled pore lining residues. Carbon, nitrogen, and oxygen atoms of amino acid residues are colored in gray, blue, and red, respectively. ECD—extracellular domain, TMD—transmembrane domain, and ICD—intracellular domain.

To narrow down possible BPC binding areas, an unbiased molecular docking-based screening approach was employed based on the recently published cryo-EM structures of the resting, open, and desensitized α7 nAChR states.^36^ Since no evidence for binding in the ECD was found in this study, this domain was excluded from the screening. Two grids were used for the molecular docking, one spanning the entire inner pore and the other one spanning the upper part of the pore and the junction to the ECD (Figure S1). While the possible binding sites of the BPCs are more localized with high convergence in both grids, QX-314 binding sites in the upper channel pore are more widely spread and oriented toward the ECD. For all three states, the conformations with the highest binding affinities of all compounds (Tables S2–S4) for each grid were found in the upper part of the channel. In a follow-up docking approach, we focused on the conformations with the highest binding energies in each grid and state in order to identify the most relevant interacting amino acid residues (results are shown in Table 4). For all three compounds, low binding affinities were found for the resting state of the receptor. For PTM0022, the calculated binding affinities were 4.01 μM, 376.7 nM, and 41.4 nM in resting, open, and desensitized states, respectively. For MB327, comparably lower affinities of 2.31 μM, 22.59 μM, and 327.80 nM were calculated for the resting, open, and desensitized states, respectively, with a remarkably low binding affinity for the open state compared to PTM0022. Noteworthy, for both BPCs, the affinity differences between the poorly bound resting state and the strongly bound desensitized state were about 70–100-fold. For QX-314, a comparably small differences (5-fold difference between highest and lowest binding affinity) in binding affinities were found between the different α7 receptor states, with the highest affinity for the open state. Interestingly, in agreement with our experimental data, all three compounds bind in the docking simulations to hydrophobic patches (yellow parts of the surface representation, Figure 5) around the highest channel ring of positively charged glutamate residues (E258, E20′, ′ indicate channel lining residue) and are positioned in different orientations, depending on the state of the α7 (Figure 5).

**Table 4:** Molecular Docking Results of the Indicated Compounds in All Three States (PDBs, See Experimental Section or Figure 5) of the α7 nAChR^a^

α7 nAChR state	compound	binding energy (kcal/Mol)	rmsd	inhibition constant (*K*_i_)	residues involved in interaction (protein chain)
resting	PTM0022	–7.36	5.15	4.01 μM	**A257** (B), A262(B), F134(C), Y209(C), **I259** (C)
	MB327	–7.69	0.9	2.31 μM	L247(A), V251(A), L247(B), V251(B), V251(C), **L254** (C), L247(D), **L254** (D), **E258** (D), **L254** (E)
	QX-314	–6.79	1.01	10.49 μM	D41(A), E44 (A), E172(A), W173(A), Y209(A), **I259** (A), K45(E)
open	PTM0022	–8.76	2.06	376.70 nM	**L254** (B), A252(B), Y209(C), **Y210** (C), N213(C), L214(C), V251(C), **L255** (C), **I259** (C)
	MB327	–6.34	2.6	22.59 μM	L45(B), **E258** (B), **Y210** (C), L214(C), **L255** (C), **I259** (C)
	QX-314	–7.72	1.95	2.18 μM	K45(B), A262(B), D41(C), V42(C), E44(C), E172(C), W173(C), **I259** (C)
desensitized	PTM0022	–10.07	0.42	41.40 nM	Y209(A), L214(A), V251(A), F252(A), **L254** (A), **L255** (A), V256(A), **E258** (A), **I259** (A), **L254** (E), **A257** (E), **E258** (E)
	MB327	–8.85	1.19	327.80 nM	L246(C), L247(C), M253(C), N213(D), L214(D), P217(D), I221(D), V245(D), F252(D)
	QX-314	–7.35	0.68	4.10 μM	M253(D), **L254** (D), **E258** (D), N213(E), L214(E), F252(E), **L255** (E)

a Compounds were docked in a grid covering interacting residues identified during the screening shown in Figure S1 and Tables S2–S4 according to the Experimental Section. Amino acid residues identified to differ between 5HT3A and α7 nAChR and mutated in this study are bold.

**Table 5:** EC_50_ Values and Hill Coefficients (*n*_H_) for the Functional Chimeras of Human α7 and Mouse 5HT3A Receptors^a^

chimera	ligand	EC_50_	(95% CI) μM	*n*_H_
α7	ACh	35.5	(32.8–38.4)	2.06
α7^V201^^–^^5HT3A^	ACh	38.6	(32.3–46.6)	1.85
α7^4TM 5HT3A^	ACh	44.1	(41.6–46.8)	2.30
α7 SDT	ACh	15.4	(12.4–19.9)	1.17
α7^Anion^	ACh	0.23	(0.19–0.27)	0.90
α7^Anion^ E258R	ACh	2.62	(2.17–3.19)	1.33

a Values in parentheses represent 95% confidence intervals (95% CI).

### Mutagenesis Supports a PTM0022 Binding Area in the Upper Part of the α7 Channel Pore

**Figure 6:**
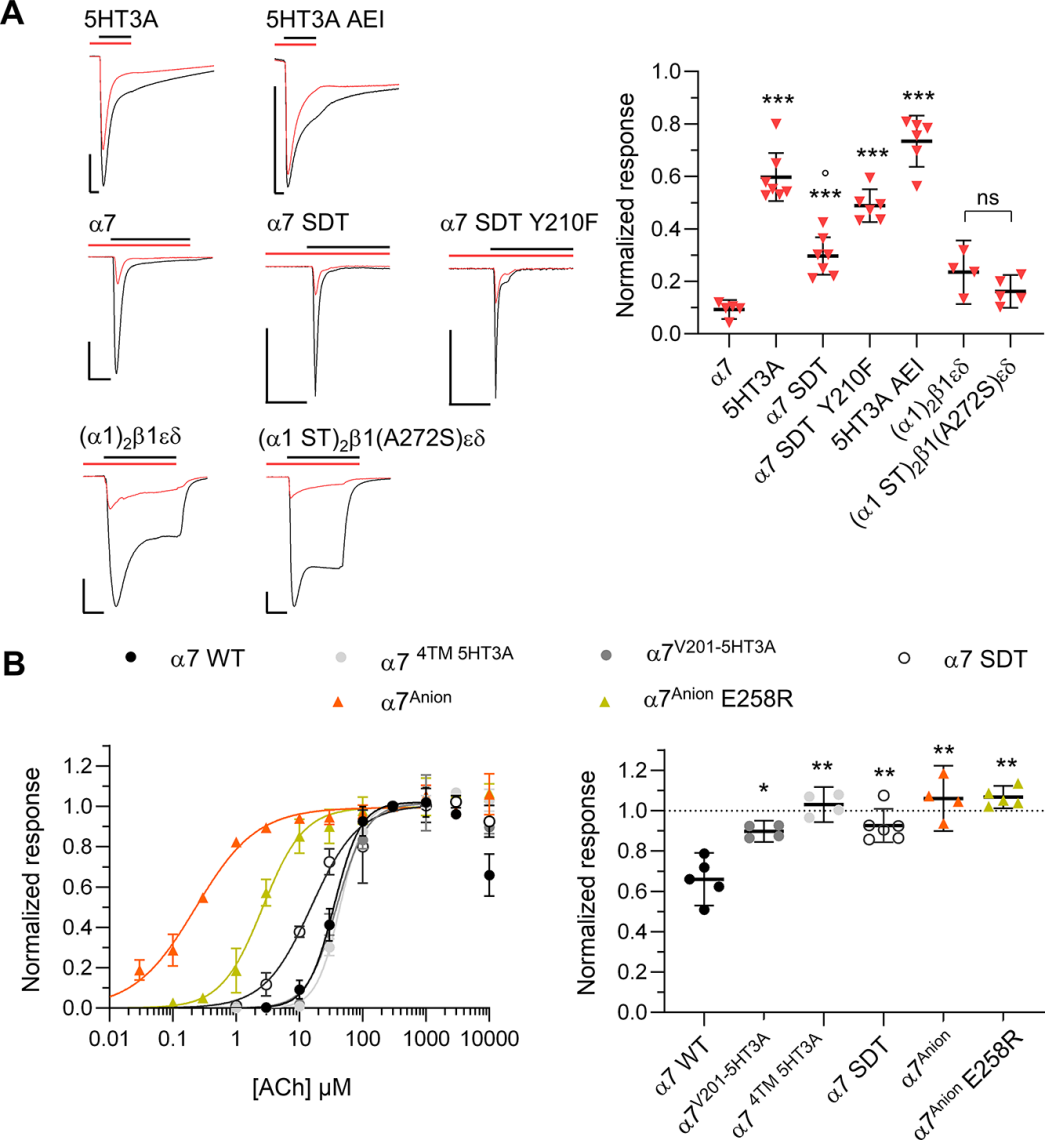
Analysis of α7 nAChR mutants to identify residues involved in the inhibition by PTM0022. (A) Representative current traces (Scale bars represent 0.5 μA and 2 s) and statistical analysis showing inhibition of the indicated α7 mutants by 1 μM PTM0022 (red triangle). For details of the mutants, refer to Table S1. Mean of the individual values is displayed as black line. Error bars represent the 95% CIs from *n* = 4–6 experiments. Statistical analysis was done using Brown–Forsythe and Welch ANOVA with posthoc Dunnett T3 test compared to α7 with *p* < 0.05 *, *p* < 0.01 **, and *p* < 0.001 ***, to 5HT3A with *p* < 0.01°. Current responses (EC_80_ agonist) were normalized to responses before—preincubation and co-application of PTM0022. (B) ACh dose response curves (EC_50_ values are in Table 5) for the indicated α7 mutants and chimeras with the mean and SD (left panel) and detailed analysis of responses to 10 mM ACh showing single values with means and 95% CIs (right panel).

Because of its high potency on both α7 and muscle-type nAChRs, PTM0022 was used to experimentally confirm the essential amino acid interactions identified in the docking simulations (Table 4). Based on a sequence alignment between 5HT3A and α7 nAChR (Figure S4), the respective α7 residues were replaced by the corresponding residues of the 5HT3A receptor, and vice versa. As seen in Figure 6A, an exchange of three amino acids, E258D, forming the negative ring, and the two neighboring residues A257S and I259T (orange frame in Figure S4) reduces the potency of 1 μM PTM0022 already significantly. This is even further reduced by an additional Y210F exchange (α7 257-259SDT, Y210F), resulting in responses similar to those at the 5HT3A receptor. The reverse substitutions in the 5HT3A receptor (SDT into AEI) did, however, not reconstitute α7-like properties, suggesting that in addition to the charged patch, a favorable channel geometry is required. Likewise, no change in inhibition was seen when the corresponding residues in the negative ring of the muscle-type α1 subunit were substituted with S257 and T258 of the 5HT3A receptor (α1 ST) in addition to β1 A272S just before the negative ring in the β1 subunit (see the orange frame in Figure S4). This indicates a distinct or more complex binding area in the heteromeric muscle-type nAChR. Interestingly, as shown in Figure 6B, the α7–5HT3A-chimeras, the α7 nAChR SDT, the anionic α7 receptor (α7^Anion^), and the α7^Anion^ E258R receptor were not inhibited by 10 mM ACh, confirming that the OCB by ACh is dependent on its charge and likely involves the same residues that are critical for inhibition by BPCs.

### Correlation between Typical Physicochemical Properties of the Investigated BPCs and Their Potency toward the α7 nAChR

**Figure 7:**
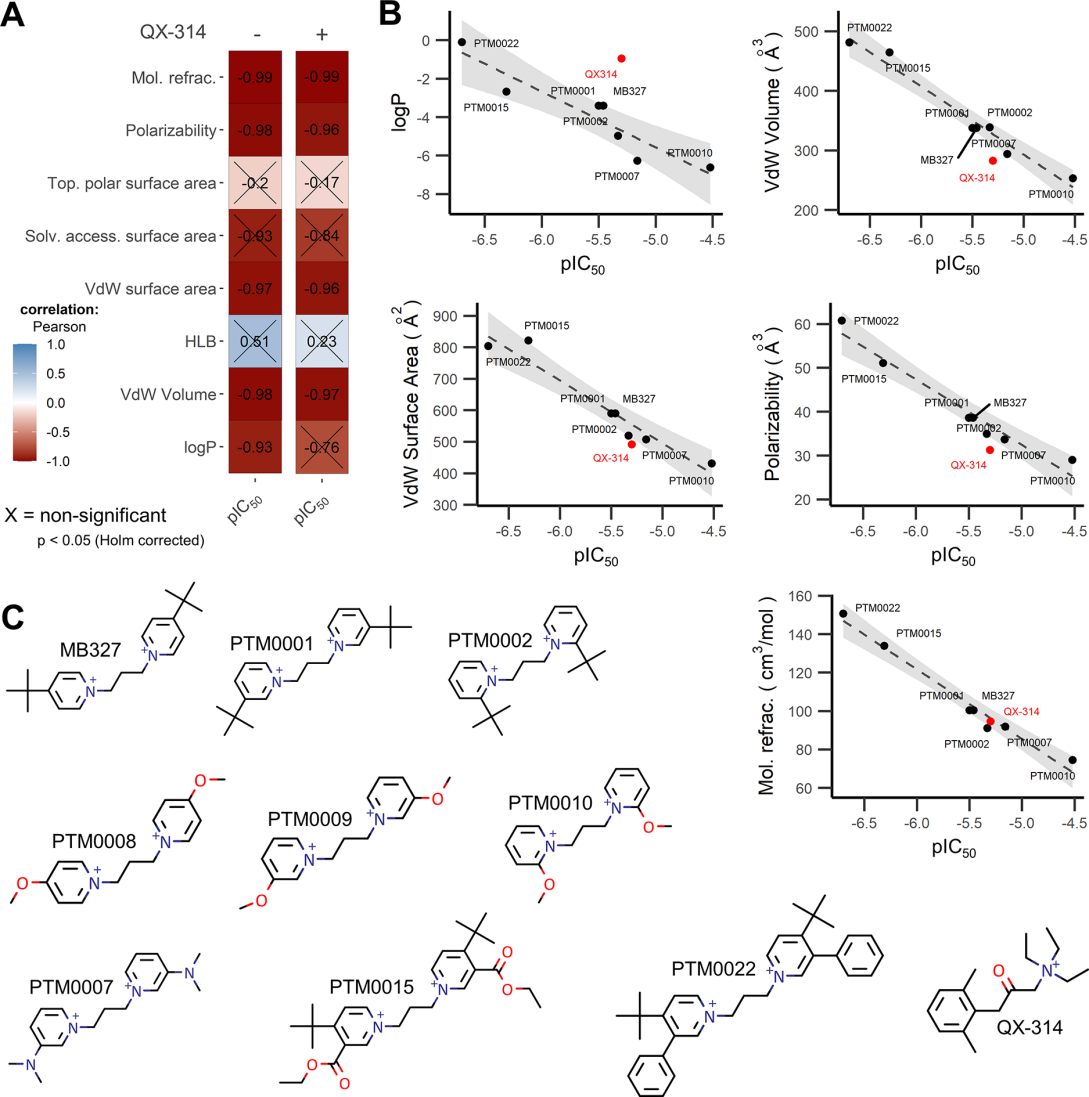
Correlation analysis of the potency of the BPCs at the human α7 nAChR and their chemical properties. (A) Pearson correlation of the determined pIC_50_ values and relevant QSAR chemical properties (see Table S5) obtained from http://chemicalize.com/ (ChemAxon) with significance threshold of *p* < 0.05 (Holm corrected) with (+, *n* = 8) or without (−, *n* = 7) QX-314. Negative and positive correlations are indicated with red and blue, respectively. (B) Scatter plots of the five significant chemical properties from A without (−) QX-314 with a smooth fit (*x*–*y*, dashed line) and 95% confidence interval (light gray) of the fit. QX-314 is shown as red point, independent from the fit. Note that pIC_50_ values could not be determined for PTM0008 and 09 and they are not included in this analysis (C) chemical structures of the symmetrical BPCs and QX-314. Oxygen and nitrogen atoms are colored red and blue, respectively. Access, accessible; mol, molar; refract, refractivity; sol, solvent; top, topological; VdW, van der Waals.

To test the hypothesis, the size of the BPC correlates with its ability to block the α7 pore, we performed a correlation analysis with the physicochemical properties of the tested BPCs and their log-transformed potencies (pIC_50_) at the α7 nAChR (Figure 7A). This revealed strong and significant correlations for the log *P*, van der Waals surface area, polarizability, van der Waals volume, and associated molar refractivity. Inclusion of the structurally unrelated QX-314, however (Figure 7C), showed a low correlation for the log *P* as a measure of the hydrophobicity (Figure 7A,B), while the van der Waals volume and the more sophisticated molar refraction and polarizability^37,38^ were still strongly correlated with a higher potency. Given the correlation of polarizability, molar refraction, and van der Waals volume among each other (Figure S7) for the given set of data, the size of the BPC is likely the driving descriptor. Supported by the fact that the linker length (as one way of altering the molecular size) was already a reported determine of their potency.^39^

### PTM0022 Is Not a Resensitizer of the α7 nAChR

**Figure 8:**
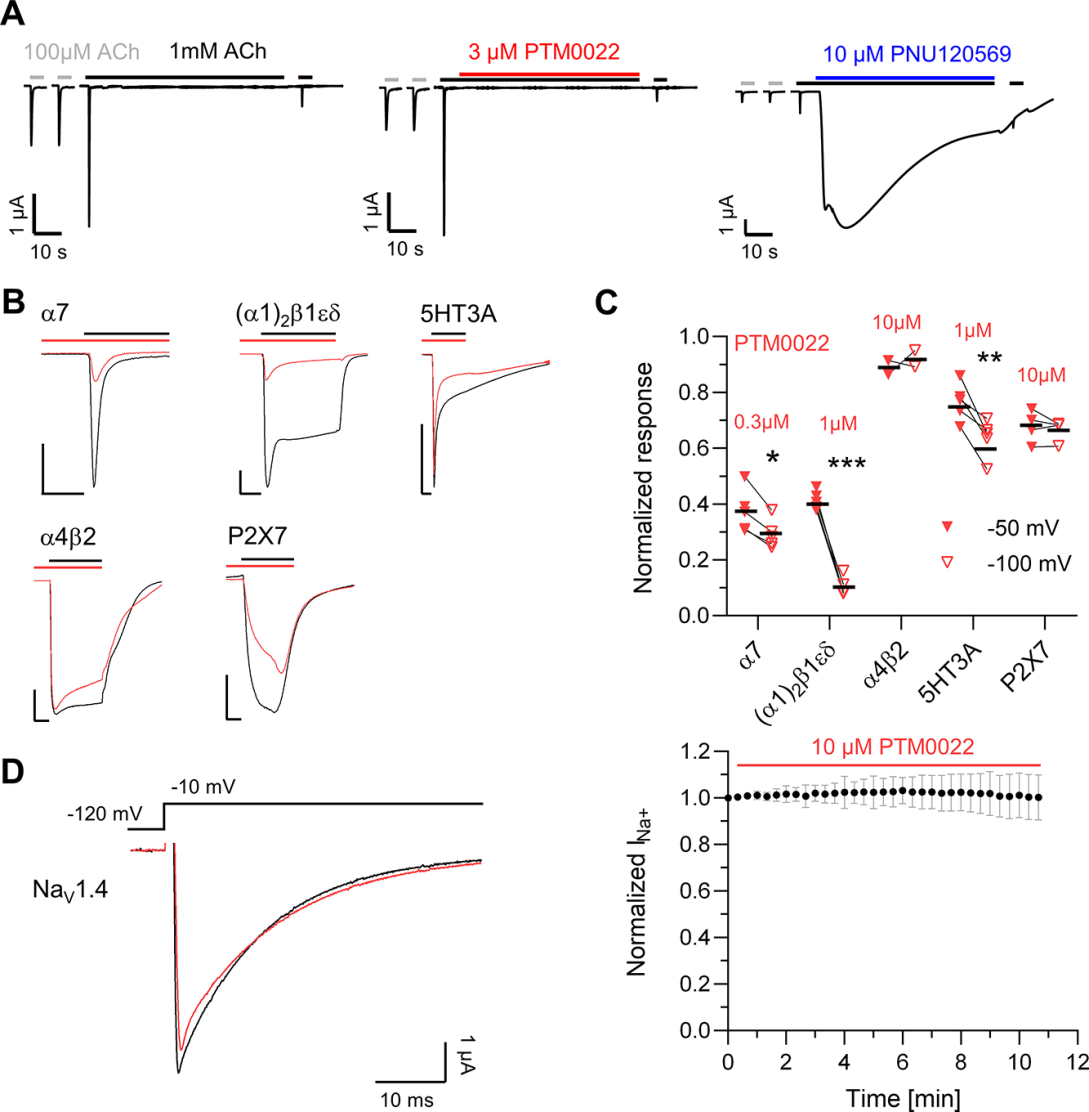
BPC do not resensitize oocyte-expressed α7 nAChR and show weak inhibition of some other cation-selective ion channels. (A) Oocytes were clamped at −70 mV, and 5 s-pulses of 100 μM ACh (gray lines) were applied in 1 min intervals until stable responses were obtained. Receptors were then desensitized by a 1 min application of 1 mM ACh (black lines) with or without 10 μM PNU120596 (blue line) or 3 μM PTM0022 (red line). After a 5 s wash-out, 1 mM ACh was reapplied. Representative current traces from three different oocytes per experiment are shown. Note that PNU120596 and PTM0022 were added 7 s after the beginning of 1 mM ACh exposure to allow complete receptor desensitization. (B) Superposition of representative current traces before (black) and after preincubation and co-application (red) of the same PTM0022 concentrations as indicated in (C) for respective ligand-gated ion channels clamped at −100 mV. Scale bars represent 0.5 μA and 2 s. (C) Voltage-dependency of the inhibition shown in (B). Single values with the mean as black line for −50 and −100 mV are shown. Paired *t*-test with *p* < 0.05 *, *p* < 0.01 **, and *p* < 0.001 ***. (D) Left panel, typical current traces of Na_V_1.4 before (black) and 12 min after (red) application of 10 μM PTM0022. Currents were elicited by 50 ms pulses to −10 mV from a holding potential of −120 mV every 20 s. Right panel, sodium peak currents normalized to the control pulse before application of PTM0022. Means and SD values of four different oocytes are shown.

BPCs have been reported to recover OPC-inhibited muscle contractility in an AChE-independent way. This was shown in guinea-pig hemidiaphragm preparations for symmetrical BPCs such as SAD-128, TMB-4,^6^ and MB-327,^10^ as well as in human muscle preparations and rat hemidiaphragm preparations for MB-327.^11^ In vivo experiments in guinea-pigs with MB399 (di(methanesulfonate) salt of MB327 with increased water solubility) confirmed a protective effect against OPC.^10,40^ BPCs were further shown to potentiate α7 currents in transfected COS cells.^20^ To further investigate the potential of BPCs to facilitate resensitization of desensitized nAChRs, we tested whether they could protect or recover the oocyte-expressed α7 nAChR from desensitization. Therefore, we desensitized the α7 nAChR for 1 min with 1 mM ACh and assessed its recovery after a 5 s perfusion with buffer with and without 3 μM PTM0022. As shown in Figures 8A and S2, no current increase was observed in the presence of PTM0022, MB327, or QX-314. Different application schemes for PTM0022 (before or together with the 1 mM ACh-stimulation) did not alter the outcome (see Figure S3). In contrast, co-application of the PAM PNU120596 caused immediate activation and potentiation of the receptor and delayed desensitization, thus providing support for the concept of PAMs as potential treatment for OPP.

### PTM0022 Is Selective for α7 and Muscle-type nAChRs

Since the investigated BPCs also showed some inhibition of the 5HT3A receptor, we tested the ability of PTM0022 to inhibit other cation-selective ion channels (Figure 8B–D). We found voltage-dependent inhibition of the 5HT3A (Figure 8C), supporting an action as an OCB. Interestingly, 10 μM PTM0022 did not inhibit the α4β2 nAChR at −50 and −100 mV, while the trimeric purinergic P2X7 receptor was slightly inhibited but in a voltage-independent way. The voltage-gated muscle sodium channel Na_V_1.4, which could be a relevant target for the treatment of the OPP, was also not affected by 10 μM PTM0022 (Figure 8D). Thus, PTM0022 appears to be rather selective for α7 and muscle-type nAChR subtypes.

## Discussion

Here, we investigated a series of BPC analogues on defined oocyte-expressed human muscle-type and neuronal α7 nAChRs and showed that they act as noncompetitive antagonists of the α7 and muscle-type nAChRs and that inhibition is voltage-dependent. The simplest interpretation of these data is that BPCs act as channel blockers. A charge-dependent binding within the upper channel pore close to the ECD was supported by mutagenesis and computational analysis.

Our findings are in agreement with several previous reports, in which the actions of the BPCs SAD-128 or MB327 were investigated by single-channel patch clamp analysis of nAChRs in frog and murine muscle cells^6,13^ as well as in CN21 cells,^10^ a human rhabdomyosarcoma cell line stably transfected with the adult ε-nAChR subunit.^41^ In contrast to another electrophysiological study^20^ that found a positive allosteric modulation of CHO cell-expressed human α7 nAChRs by BPCs, we did not see PAM-like effects. The reason for this discrepancy is not clear, but different ion channel properties in mammalian and frog plasma membranes could account for these findings. For example, many ion channels are allosterically modulated by membrane lipids,^42,43^ and even small changes in the protein^44−46^ or modulator^47^ can strongly alter channel properties. Also, different ligands can induce potentiating, inhibiting, or silent effects via the same allosteric binding sites.^47,48^ However, all tested BPCs showed a similar inhibitory behavior on human α7 and muscle-type nAChRs (Figure 1) as well as on the 5HT3A receptor, indicating that their mode of action is rather unspecific and tolerant to differences in the binding sites. To definitely exclude an effect of the expression system, we confirmed the antagonistic properties of MB327 by patch clamp analysis of CHO cells stable transfected with the human α7 nAChR (Figure S8). For the detailed analysis, we focused on the α7 nAChR due to the weak effects of the BPCs on the muscle receptor and the lack of known PAMs of this receptor.

PNU120596 is an α7-selective PAM with a known-binding site^25,49^ and a well-studied mode of action.^19^ To investigate a potential negative allosteric mechanism of the analyzed BPCs via the PNU120596 binding site (as suggested by the PNU-like potentiation observed for MB327 and other BPC^20^), we co-applied them with PNU120596. Based on the clearly faster (un)binding kinetics of PTM0022 compared to the kinetics of channel closure and opening caused by PNU120596 alone (Figure 2) as well as the complete recovery of the PNU120596 effect upon PTM0022 removal, we excluded a direct competition of both compounds at the allosteric PNU120596 binding site. However, these findings cannot exclude binding of MB327 to another (negative) allosteric site that modulates PNU120596 binding. Interestingly, when equipotent concentrations of PTM0022, MB327, and OX-314 were applied together with PNU120596 (compare Figure 1B), MB327 showed a clearly weaker inhibition of the potentiated open state, and QX-314 was completely unable to block the PNU120596-potentiated channel. A possible explanation is that the PNU120596-potentiated open channel state differs significantly from the nonpotentiated channel, and the larger PTM0022 more efficiently occludes the potentiated state. This assumption is supported by the correlation analysis (Figure 7), which shows that BPCs with a larger van der Waals volume mediate a stronger inhibition. While no structure of the short-lived nonpotentiated α7 open state is available, functional data indicate that both states differ in terms of subconductance levels,^19^ inward rectification,^50^ Ca^2+^ permeability,^51^ and sensitivity to channel blockers.^52,53^ Alternatively, MB327 binding might allosterically interact with the PNU120596 binding site. This would also explain the observed potency increase of MB327 at the M253L PNU120596 binding site mutant (Figure 2) and is supported by our docking results, which show close proximity of the respective binding sites (Figure 5).

Like most channel blockers, the BPCs used in this study are charged^31,54^ and showed increased inhibition at lower membrane potentials that was most pronounced at the muscle-type nAChR.^55^ While a clear differentiation between sequential^29^ and trapped^56^ binding modes requires single channel recordings, the fast washout of the BPCs (Figure S6), the voltage-dependent inhibition (Figure 2), and the ability to block other cation-selective ion channels (Figure 8B–D) support the previously proposed sequential channel block.^6^

The α7 nAChR is one of the best-studied nAChRs and high-resolution structures of antagonist-bound and apo-resting states, PNU-120596-bound open state, and epibatidine-bound desensitized states are available.^36,49^ The structure of its dynamic ICD has recently been determined by a combination of NMR, ESR, and computational approaches.^57^ Together with its homomeric structure, the detailed structural information provides a basis for mutagenesis and the generation of chimeras with the highly homologous 5HT3 receptor, which allowed us to experimentally investigate possible binding regions of the BPCs.

The binding site of QX-314 has been experimentally localized around L9′ (L247, ′ indicates pore lining residues) and T6′ (T244)^29^ in the lower part of the α7 channel pore. In the α7 nAChR, BPCs show a weaker voltage-dependency than QX-314 (Figure 3), suggesting that they may localize more toward the α7 ECD and to a deeper binding site in the muscle-type. However, based on radioligand binding assays with MB327 on purified *T. californica* membranes^14^ and docking and molecular dynamics simulations on a homology model of the human muscle nAChR,^15,16,58^ competitive binding to the orthosteric binding site and allosteric binding sites in the extracellular channel vestibule were proposed for different BPCs. In agreement with a binding site within the α7 TM region, we show that the potency of BPCs and QX-314 is clearly reduced at a α7–5HT3 chimera, in which the α7 TM and ICD domains were replaced with the respective 5HT3 sequences (α7^V201–5HT3A^, Figure 4). Interestingly, the inhibition of this chimera by QX-314 and MB327 was increased again if the α7-ICD was reintroduced. A possible explanation is that the ICD influences gating via the adjacent TM3 and 4 domains, as previously shown.^57^ Since we found MB237 localized a bit closer toward the channel gate, it might be more affected by the ICD-dependent reorientation of the TMDs, in particular TMD3 with its prolonged cytosolic helix.

At the triple mutated anionic, α7 (α7^Anion^) nAChR^32^ inhibition by MB327 and OX-314 was abolished (Figure 4), while the inhibition by the more potent PTM0022 was incomplete, and an additional E258R mutation was required for complete inhibition. This is in agreement with the role of the mutated residues in cation conductance^59^ and their involvement in binding the positively charged PTM0022, as identified by computational modeling (Figure 4).

According to the docking studies (Figure 5), MB327, PTM0022, and QX-314 (Figure S1) show the highest affinities in the transition zone of the ECD to the TMD (Tables S2–S4), where all three molecules are placed within a ring of negatively charged amino acid residues formed by E258 (E20′) and are stabilized by lipophilic and π-cation interactions. Interestingly, PTM0022 and MB327 bind close to the PNU120596 binding pocket^25,49^ and showed the highest binding affinity in the desensitized state. The poor binding affinity of MB327 in the open state (Table 4) could explain its weak effect in the PNU120596- prolonged open state. However, limitations of this open state structure^36^ and likely differences to the PNU120596-free open state need to be considered. The identified binding area at the ECD/TMD border, is in close proximity to one of the MB327 binding sites identified in the computational simulations with the *Torpedo* nAChR^58^ and human muscle-type nAChR.^16^ However, static molecular docking experiments as performed in this study are simplified since BPCs seem to compete as big cations with the small conducting cations, and the conductances and polarizability of the receptor have been neglected.^60^

The symmetrical BPCs are derived from bispyridinium oxime reactivators of acetylcholine esterase, such as obidoxim and HI-6. Various nonreactivating effects of these compounds on cholinergic neurotransmission, including modulation of presynaptic ACh content and release as well as pre- and postsynaptic muscarinic receptors, have been described,^61,62^ and BPCs may therefore be considered as “dirty drugs”. While it has been difficult to correlate *in vivo* and *in vitro* findings from different models, the nAChR-modulating effect seems to have a major contribution^62^ and the OCB action/potency correlated well with their ability to relieve tetanic block.^6^ In contrast to competitive blockers of muscle-type nAChRs, which are considered to have a narrow therapeutic window due to excess antagonism, noncompetitive antagonists like OCBs have been suggested as a practical alternative approach as their action is not overcome by increasing ACh concentration and the block is use-dependent.^63^ A mechanism as OCB is in good agreement with the positive charge of the BPCs and could also account for observed protective *in vivo* effects.^6^ However, the α7 nAChR might not be a good model to reproduce the *in vivo* action due to its very different desensitization kinetics compared to the muscle receptor.

## Conclusions

Allosteric ion channel modulation has great therapeutic potential.^18^ In the case of the OPP, it has been hypothesized that resensitization of desensitized muscle-type nAChRs can counteract OPC-induced respiratory failure and other nAChR-induced symptoms. This principal concept is supported by the resensitization of a completely desensitized α7 nAChR by PAMs like PNU120596.^20^ However, no PAM acting at the muscle-type nAChR, a major target in the OPP, has been described so far, and therefore, this concept could not be validated *in vivo*. Moreover, it needs to be considered that PAMs can alter receptor kinetics, which might severely influence physiological muscle function. Use-dependent channel blockers that decrease the ion flux during a burst without altering the kinetic channel properties of the ion channel are already employed clinically as local anesthetics, antiarrhythmics, and antiepileptics for other ion channels^64,65^ and could offer a practical therapeutic alternative to counteract ACh-induced depolarization block at the neuromuscular junction.^63^ Our functional data do not support a PAM effect of BPCs but confirm previous studies^6,10,13^ that demonstrated that symmetrical and nonsymmetrical BPCs act as OCBs. In addition, we provide a model for their binding mechanism, which might serve as a basis for the development of more potent and specific compounds.

## Experimental Section

### Materials

Components of buffers and serotonin hydrochloride were obtained from Carl Roth, Germany. Acetylcholine (ACh), QX-314, and MLA were obtained from Sigma-Aldrich and Merck Eurolabs, Germany, and PNU120596 was obtained from Tocris Bioscience, USA. All chemicals were purchased in the highest available purity.

MB327 was synthesized by the Defense Science and Technology Laboratory (Dstl), Porton Down, Salisbury,^66^ and PTM BPCs were synthesized in the group of Prof. Dr. Wanner (Department of Pharmacy—Center for Drug Research, Ludwig-Maximillians-Universität München, Germany) in purities of ∼98%.^20,21^ MB327 and PTM compounds were kindly provided by Karin V. Niessen and Thomas Seeger.

Stock solutions were prepared in ND96 (see below) or, in the case of PNU120596, in DMSO and kept in aliquots at −20 °C until use.

### Frogs and Oocyte Preparation

*X. laevis* females were obtained from Nasco (Fort Atkinson, WI, USA) and kept at the core facility animal models (CAM) of the biomedical center (BMC) at the LMU Munich (Az:4.3.2–5682/LMU/BMC/CAM) in accordance with the EU Animal Welfare Act. To obtain oocytes, frogs were anesthetized with MS222, killed by decapitation, and the ovary was surgically extracted. In some cases, oocytes were provided by Prof. Luis Pardo (Max-Planck-Institute for Multidisciplinary Sciences, Göttingen) or Ecocyte Bioscience (Castrop-Rauxel, Germany). Ovaries were dissociated in 2 mg/mL collagenase (NB 4G proved grade, Nordmark Pharma GmbH) in ND96 (96 mM NaCl, 2 mM KCl, 1 mM CaCl_2_, 1 mM MgCl_2_, 5 mM HEPES, and pH 7.4), and defolliculated by gentle shaking in Ca^2+^-free ND96 (about 20 min at RT), and kept at 16 °C in filtered ND96 supplemented with 5 μg/mL gentamicin.

### cDNAs, Cloning, Mutagenesis, and cRNA Synthesis

cDNA for human adult muscle-type nAChR subunits α1, β1, ε, and δ in the pT7TS vector and the human α7 subunit in the pMXT vector (originally from Jon Lindstrom, University, Pennsylvania, PA, USA) was a gift from Prof. David Adams (Illawara Health and Medical Research Institute, Wollongong University, Australia). cDNAs of human α4 (L35901.1, with silent base exchanges to reduce GC content) and β2 (X53179.1) nAChR subunits, mouse 5HT3A (M74425.1,^67^), human NACHO/TMEM35A (NM_021637.3), and a short fragment carrying a P237 insertion and E237A and V251T point mutations (to generate α7^Anion^, see Table S1)^32^ were synthesized (Genewiz, Azenta Life Science, USA) and cloned into the pNKS2 vector^68^ by Gibson assembly^69^ using Q5 polymerase and reagents from New England Biolabs (USA). Receptor chimeras and point mutations were created by Gibson Assembly or site-directed mutagenesis [KLD Enzyme Mix, New England Biolabs (USA)]. See Table S1 for details about the receptor chimeras and mutants. Oligonucleotides were from metabion international AG, Germany. All constructs were confirmed by sequencing of the whole cDNA (Eurofines Genomics, Germany). Plasmids were linearized using *NotI* (pNKS2), *Bam*HI (pMXT), or *XbaI* (pT7TS) from New England Biolabs (USA), and cRNA was synthesized using the mMessageMachine kit (Invitrogen, Thermo Fisher Scientific, USA).

Rat NaV1.4 in pcDNA3.1 (Uniprot: P13390(70)) was kindly provided by Stefan Heinemann, University of Jena. Human P2X7 was used, as described in ref (71).

### Electrophysiological Recordings

If not otherwise noted, stage IV *X. laevis* oocytes were injected with 50 nL aliquots of cRNA (500 ng/μL). cRNA of the α7 SDT mutant (see Table S1) was coinjected with NACHO cRNA (ratio 1:1). Reduced cRNA concentrations were injected for α7, (α1)_2_β1εδ (2:1:1:1 subunit ratio), 5HT3A (100 ng/μL), the 5HT3A AEI mutant (see Table S1) (20 ng/μL), and the α7^4TM 5HT3A^ chimera (4 ng/μL). 23 nL of Na_V_ 1.4 cDNA (150 ng/μL) were injected into the nucleus.^72^

1–5 days after injection, TEVC recordings were performed in ND96 at a holding potential of −70 mV, unless otherwise stated. P2X7 measurements were performed at −60 mV in ORI (90 mM NaCl, 1 mM KCl, 1 mM CaCl_2_, 1 mM MgCl_2_, 5 mM HEPES, and pH 7.4), and ATP was applied in ORII (90 mM NaCl, 1 mM KCl, 2 mM MgCl_2_, 5 mM HEPES, and pH 7.4). The voltage-gated sodium channel Na_V_ 1.4 was held at a membrane potential of −80 mV and activated by a 50 ms pulses of −10 mV, preceded by a 50 ms pulse of −120 mV. Microelectrodes were pulled from borosilicate glass, filled with 3 M KCl, and had resistances between 0.3 and 1 MΩ. Membrane currents were recorded using a Turbo Tec 05X amplifier (npi electronic, Tamm, Germany), filtered at 200 Hz, and digitized at 400 Hz using CellWorks software (npi electronic, Tamm, Germany). Currents were filtered at 3 kHz and digitized at a sampling frequency of 10 kHz. Solutions were automatically applied with a VC3–8xP valve system (ALA scientific instruments, USA), and perfusion speed was regulated by air pressure using an PR-10 analog pressure regulator (ALA scientific instruments, USA). Oocytes were placed in a 200 μL Teflon bath (Automate Scientific, USA), and solutions were applied via 1 mm dimeter Teflon tubing and a Teflon micromanifold to minimize ligand binding to surfaces. Oocytes were continuously perfused at 20 μL/s, and ligand-containing solutions were applied at ∼250 μL/s. All measurements were performed with oocytes from at least two different frogs.

### Recording Protocols

To determine agonist dose response relations, stable current responses were established by application of 5 s pulses of a reference concentration (300 μM ACh for wt α7 nAChR and all mutants and chimeras, except for α7^Anion^ and α7^Anion^ E258R, which had a higher ACh sensitivity and were activated with 10 μM ACh) in 2 min intervals. Then, the reference concentration and increasing ACh test concentrations were alternatingly applied. In the case of low test concentrations, an additional 2 s pulse of reference concentration was applied immediately after the test concentration to maintain equal fractions of desensitized channels. Each test concentration was normalized to the mean of the preceding and following reference concentrations. To investigate competitive antagonist effects, agonist solutions were supplemented with a constant concentration of antagonist, and responses were normalized to the reference concentration without antagonist.

To determine current inhibition in the presence of antagonists and for antagonist dose response curves, 7 s pulses of the following agonist concentrations were used: 100 μM ACh for all α7 nAChR receptors (including mutants and chimeras), 30 μM ACh for muscle-type nAChR, 5 μM 5HT for 5HT3A (including mutants and chimeras), and 300 μM ATP for P2X7. Agonists were applied in intervals of 2 min with perfusion plus 20 s without perfusion. After reproducible current responses were obtained, oocytes were perfused for 2 min with buffer, and then the antagonist was perfused for 3 s and preincubated for 17 s in a static bath before co-application of the agonist and antagonist. Current responses in the presence of an antagonist were normalized to the last agonist response before antagonist application.

In the case of PNU120596/antagonist co-application, responses to ACh (100 μM) were stabilized at 2 min and 20 s intervals, as for the antagonist does response curves. Once stable currents were obtained, 10 μM PNU120596 was perfused for 3 s with a static bath for 17 s. Subsequently, ACh and PNU120596 were coapplied for 5 s, followed by a 9 s application of additional antagonist, followed by another 10 s of only ACH and PNU120596.

To investigate α7 resensitization by different ligands, stable current responses were established with pulses of 100 μM ACh at 2 min intervals. Then one 5 s pulse of 1 mM ACh was applied, and solution flow was stopped for 1 min, followed by 5 s perfusion with buffer and another 5 s pulse of 1 mM ACh (control). In the case of PTM0022 or PNU120596 application (test), these were coapplied with 1 mM ACh 7 s after application of only 1 mM ACh. Note that PNU120596 was continuously perfused to avoid issues with current stability during the recording. Control and test responses were recorded from the same oocyte at 5 min intervals of buffer perfusion.

### Data Analysis and Visualization of Electrophysiological Recordings

Recordings were imported from CellWorks into Clampfit 11 (Molecular Devices, pClamp, RRID:SCR_011323), baseline corrected, and analyzed. In the case of small currents, recordings were digitally low-pass filtered at 20 Hz to eliminate the noise. To reduce errors due to the fast desensitization of the α7 nAChR, net currents [=areas under the curve (AUCs), Meyer et al., 1998] were analyzed instead of current amplitudes. Dose–response analyses were performed with GraphPad Prism version 9 (RRID:SCR_002798), and dose–response curves were fit to the data using the inbuilt Hill equation normalized response = (bottom + (top-bottom))/(1 + (IC_50_^*n*_H_/c^)) for antagonists and normalized response = (bottom + (*c*^*n*_H_^ × (top-bottom)))/(*c*^*n*_H_^ + EC_50_^*n*_H_^) for agonists with *n*_H_ = Hill slope, *c* = concentration (μM), and bottom and top constraint to 0 and 1, respectively.

Current traces were plotted with R (R Project for Statistical Computing, RRID:SCR_001905) using the following packages: dplyr (RRID:SCR_016708), tidyr (RRID:SCR_017102), and ggplot2 (RRID:SCR_014601). In the case of averaged current traces, recordings of the currents prior to antagonist exposure (control) and with the antagonist (test) of five oocytes were averaged. Each current trace was first baseline corrected, and then all data points of the test current were normalized to the peak current of the respective control current. The mean and standard deviation of each time point were calculated. Note that, due to this normalization, absolute current values cannot be shown.

### Molecular Docking and Sequence Alignment

3D-structures of PTM0022, MB327, and QX-314 were generated with MarvinSketch 21.3 (ChemAxon, RRID:SCR_004111) and saved as pdb files. Cryo-EM structures of the bungarotoxin-bound resting, PNU120596- and epibatidine-bound open, and epibatidine-bound desensitized states of human α7 nAChR were obtained from the Protein Data Bank (PDB, RRID:SCR_012820, PDB IDs: 7KOO, 7KOX, and 7KOQ, respectively^36^). Receptor structures were cleared of glycosylation, ligands, and water molecules using PyMol (RRID:SCR_000305, Schrödinger), and receptor and ligand structures were prepared with AutoDockTools 1.5.7.^73^ Hydrogen atoms (merged nonpolar hydrogens) were added, and the Boltzmann and Gasteiger Charges^74^ were computed. The ligands were kept flexible with 6, 8, and 7 rotatable bonds for MB327, PTM0022, and QX-314, respectively. 3D grids of the noncovalent interactions between the prepared ligand and receptor states, as well as electrostatic potentials and desolvation-free energies, were mapped with AutoGrid4 (RRID:SCR_015982). In a virtual screening approach, two grids (*X* × *Y* × *Z*, spacing 0.375 Å), one covering the transition area between the extracellular and transmembrane domains with 90 × 90 × 70 Å and one covering the whole channel pore with 60 × 60 × 126 Å (see Figure S1), were used to mainly cover binding pockets accessible from within the channel pore. Molecular docking was performed with autodock4 (RRID:SCR_012746) (Huey et al., 2007) using the lamarckin genetic algorithm (LGA) with 50 GA runs, a population size of 200, and 2,500,000 maximum numbers of evaluations (remaining parameters with default settings). The resulting 50 possible conformations per grid were ranked by the lowest binding energy (see Tables S2–S4 and Figure S1), and the convergence of the confirmations was analyzed by hand. For conformations with the lowest binding energy, the interacting amino acid residues of the receptor were analyzed with the Protein–Ligand Interaction Profiler (PLIP^75^), and a final docking was performed using a grid of 60 × 60 × 60 Å around these identified amino acid residues with 10 GA runs, a population size of 300, and 25,000,000 maximum numbers of evaluations. The interacting amino acid residues were again analyzed with PLIP and visualized with PyMol. For surface representation of hydrophobic interactions, the YRB script by ref (76) and Inkscape (RRID:SCR_014479) were used. In cases where PNU120596 is shown in PDB ID 7KOX (Figure S1), it was aligned with the structure in PDB ID 7EKT (using PyMol), the published cryo-EM structure of the human α7 with covisualized PNU120596.^49^ Sequence alignments were performed with Jalview (RRID:SCR_006459) using the muscle algorithm^77^ with default settings. The canonical amino acid sequences were taken from the Uniprot database (Universal Protein Resource, RRID:SCR_002380).

### Correlation Analysis

The physiochemical properties of the BPCs were calculated using the chemicalize service by ChemAxon (RRID:SCR_004111). The correlation analysis was performed using R and the ggplot2-based ggcorrmat function of the ggstatsplot package. The physiochemical properties were correlated with the log-transformed IC_50_ (pIC_50_) determined in this study (Table 1). The correlation analysis used the person correlation and Holmes-corrected significance analysis with α = 0.05.

## Supporting Information Available

Details of investigated α7/5HT receptor mutants and chimeras and their functionality; three tables with docking parameters for each grid in each receptor state for MB327, PTM0022, and QX-314; additional molecular docking results; two figures showing resensitization experiments with PNU120596 and PTM0022; MB327 and QX-314; multisequence alignment of relevant cys-loop receptors; selected chemical properties of all used BPC compounds for correlation analysis; analysis of the effect of NACHO-*co*-expression α7 nAChR mutant on potency of PTM0022; recovery of nACh-induced response after washout of BPCs from α7 and muscle nAChR; full multivariant correlation analysis for the BPCs; and manual patch-clamp analysis of the MB327 effect on α7 nAChRs expressed in CHO cells and corresponding methods (PDF)
