# Supplementary material for: Symmetrical Bispyridinium Compounds Act as Open Channel Blockers of Cation-Selective Ion Channels
Source: ACS Pharmacol Transl Sci. 2024 Feb 15;7(3):771–86. doi: 10.1021/acsptsci.3c00308 (PMC10941285; doi:10.1021/acsptsci.3c00308)
Supplement: Supplementary file 1 — pt3c00308_si_001.pdf [file pt3c00308_si_001.pdf]

Supporting Information

Symmetrical bispyridinium compounds act as open channel blockers of cation-selective ion channels

Yves Haufe[a], Dominik Loser[b], Timm Danker[b], Annette Nicke[a*]

[a] Walther Straub Institute of Pharmacology and Toxicology, Faculty of Medicine, LMU Munich, Munich, Germany
[b] NMI Natural and Medical Sciences Institute at the University of Tübingen, 72770 Reutlingen, Germany.

*** Correspondence:**
Annette Nicke: annette.nicke@lrz.uni-muenchen.de

Table S1: 5HT3/α7 receptor chimeras used in this work and representative current traces recorded from *X. laevis* oocytes. Mouse 5HT3A receptor is shown in dark grey, human α7 nAChR in light grey. Chimeras marked with × were not functional. If accessory proteins were co-expressed, they are indicated below the name of the respective construct. Amino acids numbers at the junction are given for the respective receptor subunits (numbering starting after a 23 amino acid long signal sequence for both subunits). In case of the P237 insertion (V), the numbering of the original receptor is shown but marked with a +1 to take into account the shift in numbering. Scale bars indicate 2 s and 0.3 μA. Acetylcholine was applied at EC_{80} (shown on top of each trace). Note that Eiselé et al. and Gee at al. have worked with rat α7 nAChR and mouse 5HT3A. Bertrand et al. worked with human constructs for both. For references, see main manuscript. α7 E258R and α7 E258A were not functional (not listed).

Construct	Schematics (1-3 TMD, 4 TMD)	Trace	Ref.
α7 V201-5HT3A	α7: V201; 5HT3A: I218	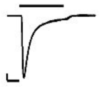	Eiselé, J. et al. 1993
α7 4TM 5HT3A	α7: V201, K303, C442; 5HT3A: I218, P318, V434	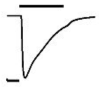	Gee, V. et al. 2007, Bertrand et al. 2008
α7 3TM 5HT3A	α7: V201, K303; 5HT3A: I218, P318	×	Gee, V. et al. 2007, functional
α7 2TMD 5HT3A	α7: A233, S264; 5HT3A: D250, T279	×	this work
α7 SDT + NACHO	α7: A257S E258D I259T; 5HT3A	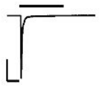	this work
α7Anion	α7: 237P V251T +1; α7: +1 E237A	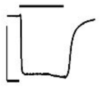	Galzi, J. et al. 1992
α7Anion E258R	α7: 237P V251T +1; α7: +1 E237A +1 E258R	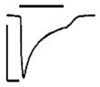	this work
α7Anion E258A	α7: 237P V251T +1; α7: +1 E237A +1 E258A	×	this work
5HT3AV217-α7	5HT3A: V217; α7: T202	×	Bertrand et al. 2008, functional
5HT3A4TM α7	5HT3A: V217, P318, Y433; α7: T202, G302, V443	×	this work
5HT3A3TM α7	5HT3A: V217, P318; α7: T202, G302	×	this work
5HT3A2TMD α7	5HT3A: P249, P277; α7: D234, M260	×	this work
5HT3A AEI	5HT3A: S273A D274E T275I; α7	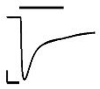	this work

Table S2: The five highest ranked dockings of *PTM0022* in the indicated states of the α7 nAChR (PDB:7KOO, 7KOX, 7KOQ) either docked in the upper part of the pore or in the channel with a grid (X,Y,Z) of 90,90,70 or 60,60,126, respectively.

nAChR α7 state	*Grid*	*Ranking*	*Binding energy (Kcal/Mol)*	*Inhibition constant (Ki)*	*Residues involved in interaction*
Resting	Upper pore	**1**	**-8.21**	**958.56 nM**	**Lys45(B), Lys45(C), Tyr210(C), Ile259(C), Tyr209(D), Tyr210(D), Glu258(D), Ile259(D)**
		2	-7.52	3.09 µM	-
		3	-7.36	4.04 µM	-
		4	-7.28	4.58 µM	-
		5	-6.55	17.83 µM	-
	Channel	1	-6.60	14.42 µM	Tyr244(C), Leu247(C), Tyr244(D), Ile243(E)
		2	-6.55	15.76 µM	-
		3	-6.41	20.02 µM	-
		4	-6.15	30.97 µM	-
		5	-6.13	32.20 µM	-
Open	Upper pore	**1**	**-9.16**	**192.52 nM**	**Leu254(B), Glu258(B), Ala262(B), Tyr209(C), Tyr210(C), Asn213(C), Val251(C), Leu255(C), Ile259(C)**
		2	-9.06	228.41 nM	-
		3	-8.99	258.47 nM	-
		4	-8.77	372.74 nM	-
		5	-8.55	536.83 nM	-
	Channel	1	-8.13	1.10 µM	Leu254(A), Ala257(A), Glu258(A), Ala262(A), Leu214(B), Val251(B), Leu255(B), Ile259(B)
		2	-8.12	1.11 µM	-
		3	-7.87	1.69 µM	-
		4	-7.18	5.43 µM	-
		5	-6.88	9.04 µM	-
Desensitized	Upper pore	**1**	**-9.78**	**68.13 nM**	**Tyr209(A), Leu214(A), Val251(A), Phe252(A), Leu254(A), Leu255(A),**

				**Val256(A), Glu258(A), Ile259(A), Leu254(E), Leu255(E), Ala257(E)**
	2	-9.55	100.28 nM	-
	3	-9.49	110.40 nM	-
	4	-9.28	157.44 nM	-
	5	-9.10	212.96 nM	-
Channel	1	-8.88	308.01 nM	Thr244(A), Ile243(B), Ile243(C), Thr244(C), Thr244(D), Ile243(E), Thr244(E)
	2	-8.53	556.19 nM	-
	3	-7.61	2.65 µM	-
	4	-6.88	9.06 µM	-
	5	-6.64	13.64 µM	-

Table S3: The five highest ranked dockings of *MB327* in the indicated states of the α7 nAChR (PDB:7KOO, 7KOX, 7KOQ) either docked in the upper part of the pore or in the channel with a grid (X,Y,Z) of 90,90,70 or 60,60,126, respectively.

nAChR α7 state	*Grid*	*Ranking*	*Binding energy (Kcal/Mol)*	*Inhibition constant (Ki)*	*Residues involved in interaction (subunit)*
Resting	Upper pore	1	-6.73	11.58 µM	Asp41(A), Val42(A), Glu44(A), Glu172(A)
		2	-6.41	19.97 µM	-
		3	-6.06	36.37 µM	-
		4	-6.03	38.30 µM	-
		5	-5.98	41.42 µM	-
	Channel	**1**	**-7.65**	**2.48 µM**	**Leu247(B), Val251(C), Leu254(C), Leu247(D), Val251(D), Leu254(D), Glu258(D), Leu247(E), Leu254(E)**
		2	-7.62	2.60 µM	-
		3	-7.61	2.64 µM	-
		4	-7.61	2.64 µM	-
		5	-7.59	2.74 µM	-
Open	Upper pore	**1**	**-6.77**	**10.96 µM**	**Leu254(B), Glu258(B), Tyr209(C), Asn213(C), Leu214(C), Val251(C), Leu255(C)**
		2	-6.68	12.67 µM	-
		3	-6.66	13.23 µM	-
		4	-6.57	15.18 µM	-
		5	-6.49	17.46 µM	-
	Channel	1	-6.75	11.26 µM	Ile243(A), Thr244(A), Leu246(A), Leu247(A), Thr244(B), Leu246(E), Leu247(E)
		2	-6.63	13.88 µM	-
		3	-6.12	32.85 µM	-
		4	-6.06	35.91 µM	-
		5	-6.02	38.84 µM	-

Desensitized	Upper pore	1	-7.08	6.48 µM	Thr250(A), Met253(A), Leu254(A), Ala262(A), Pro217(B), Leu255(B), Ile259(B)
		2	-6.71	12.07 µM	-
		3	-6.64	13.54 µM	-
		4	-6.63	13.90 µM	-
		5	-6.58	15.05 µM	-
	Channel	**1**	**-8.17**	**1.03 µM**	**Ile243(C), Leu246(C), Thr250(C), Leu254(C), Asn213(D), Pro217(D), Leu224(D), Val245(D)**
		2	-5.93	44.63 µM	-
		3	-5.88	49.18 µM	-
		4	-5.79	56.71 µM	-
		5	-5.76	59.64 µM	-

Table S4: The five highest ranked dockings of *QX-314* in the indicated states of the α7 nAChR (PDB:7KOO, 7KOX, 7KOQ) either docked in the upper part of the pore or in the channel with a grid (X,Y,Z) of 90,90,70 or 60,60,126, respectively.

nAChR α7 state	*Grid*	*Ranking*	*Binding energy (Kcal/Mol)*	*Inhibition constant (Ki)*	*Residues involved in interaction (subunit)*
resting	Upper pore	1	-6.79*	10.47 µM	Val256(C), Ala257(C), Ala275(C), Leu212(D), Asn213(D), Ile216(D)
		2	**-6.34**	**22.41 µM**	Glu44(A), Glu172(A), Tyr210(A), Ile259(A), Lys45(E), Ala262(E)
		3	-6.32	23.34 µM	-
		4	-6.18	29.46 µM	-
		5	-6.13	32.24 µM	-
	Channel	1	-6.28	24.74 µM	Thr244(A), Leu247(A), Ile243(B), Thr244(B), Leu247(B), Ile243(C), Leu247(C), Leu247(D), Thr244(E), Leu247(E)
		2	-6.18	29.50 µM	-
		3	-6.12	32.56 µM	-
		4	-6.03	37.81 µM	-
		5	-6.01	39.27 µM	-
Open	Upper pore	1	**-7.36**	**4.00 µM**	Lys45(B), Pro261(B), Asp41(C), Val42(C), Glu44(C), Glu172(C)
		2	-7.11	6.16 µM	-
		3	-7.01	7.25 µM	-
		4	-6.98	7.65 µM	-
		5	-6.88	9.10 µM	-
	Channel	1	-6.27	25.31 µM	Leu254(A), Tyr209(B), Tyr210(B), Leu214(B), Phe252(B), Leu255(B), Glu258(B)
		2	-6.23	27.13 µM	-
		3	-6.20	28.60 µM	-
		4	-6.16	30.28 µM	-
		5	-6.14	31.82 µM	-

Desensitized	Upper pore	1	**-7.25**	4.85 µM	Met253(D), Leu254(D), Glu258(D), Asn213(E), Leu214(E), Phe252(E), Leu255(E)
		2	-7.21	5.17 µM	-
		3	-7.05	6.84 µM	-
		4	-6.92	8.46 µM	-
		5	-6.92	8.46 µM	-
	Channel	1	-6.87	9.23 µM	Glu237(A), Glu237(B), Glu237(C), Ser240(C), Ile243(C), Glu237(D), Thr244(D), Glu237(E)
		2	-6.70	12.17 µM	-
		3	-6.51	16.80 µM	-
		4	-6.40	20.19 µM	-
		5	-6.26	25.90 µM	-

Table S5: Relevant chemical properties for QSAR studies of the symmetrical C3-linker bispyridinium compounds obtained from chemicalize.com by chemaxon.
Abbreviations: access., accessible; mol., molar; refract., refractivity; sol., solvent; top, topological; VdW, Van der Waals.

Compound	*pIC_{50} (M)*	*logP*	*VdW Volume ($Å^3$)*	*HLB*	*VdW surface area ($Å^2$)*	*Sol. access. surface area ($Å^2$)*	*Top. polar surface area ($Å^2$)*	*Polariza-bility ($Å^3$)*	*Mol. refrac. (mol/cm^3)*
MB327	-5.46	-3.40	337.82	1	589.94	633.51	7.76	38.62	100.34
PTM0001	-5.50	-3.40	337.8	1	589.92	632.26	7.6	38.63	100.34
PTM0002	-5.33	-4.97	338.91	1	519.24	544.2	7.76	34.94	91.09
PTM0007	-5.16	-6.27	294.04	0.88	507.12	617.18	14.24	33.65	91.87
PTM0008	> -4.50	-6.80	252.99	4.43	430.54	564.5	26.22	29.02	75.94
PTM0009	> -4.50	-6.80	253.11	4.43	430.45	560.89	26.22	29.03	75.64
PTM0010	~ -4.30	-6.62	253.23	4.43	430.72	529.55	26.22	29.03	74.44
PTM0015	-6.31	-2.68	464.75	1.88	822.09	822.09	60.36	51.09	133.89
PTM0022	-6.70	-0.10	481.16	1	803.89	800.12	7.76	60.8	150.61
QX314	-5.30	-0.96	282.68	19.84	491.5	446.74	29.1	31.26	94.59

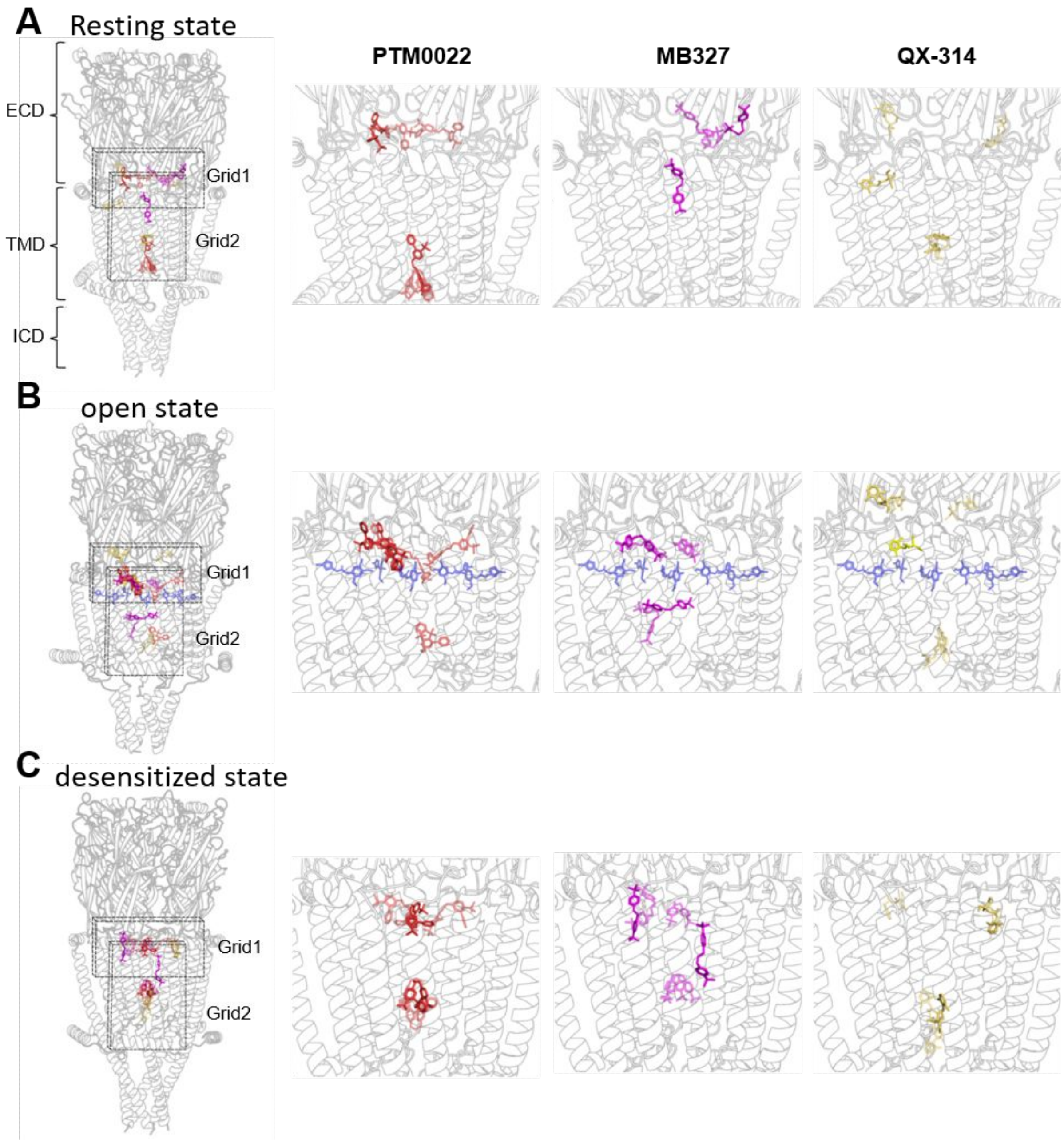

Figure S1: Molecular docking results of a screen for potential binding sites at the α7 nAChR. Highest ranked three binding positions for the shown compounds (PTM0022 - red, MB327 – magenta, QX-314 - gold) in either of the two grids (Grid1 – upper channel pore, 90x90x70 (XxYxZ); Grid2 – channel pore, 60x60x126; spacing 0.375 Å) in the resting (**A**), open (**B**) and desensitized (**C**) state of the α7 nAChR (with the highest ranked ones being more intensively colored). Refer to experimental procedure for docking parameters. PNU120596 (blue) is shown for orientation (based on PDB 7EKT (Zhao *et al.* 2021)).

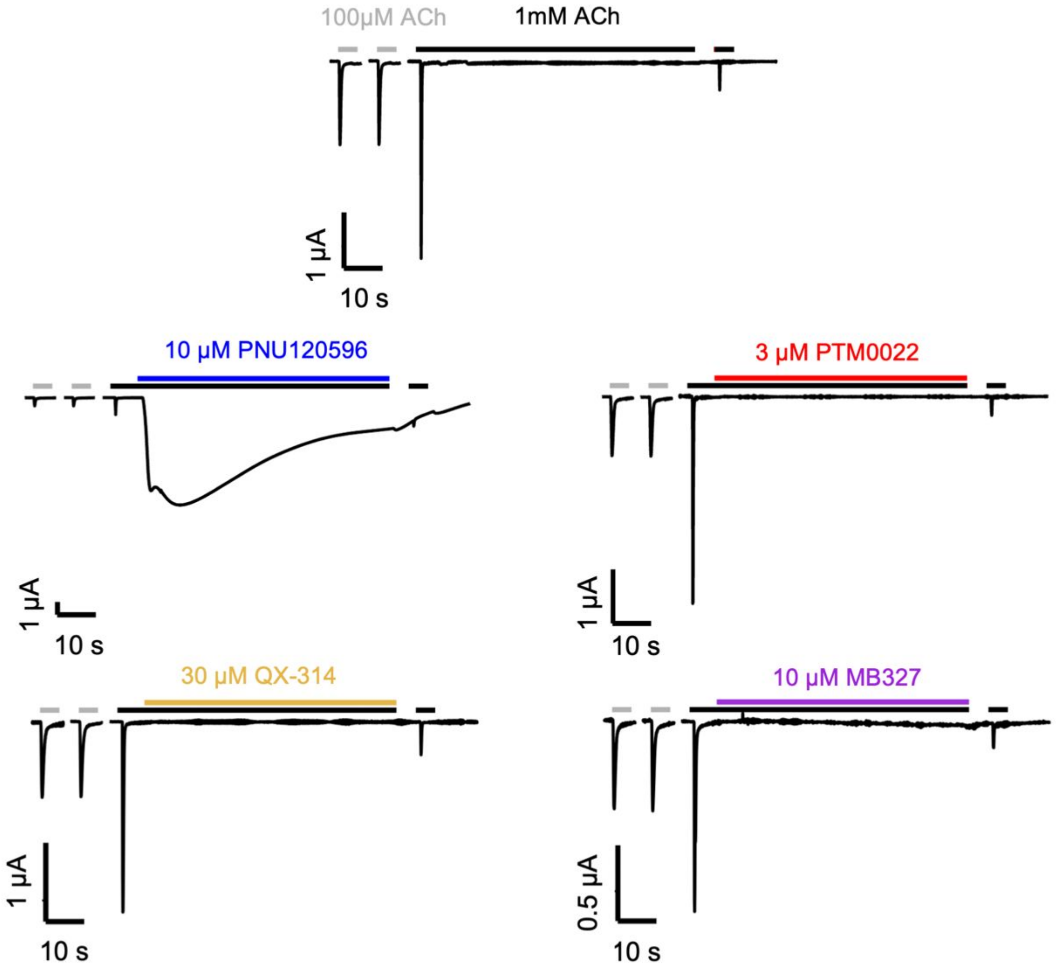

Figure S2: Application of open channel blockers does not recover receptors from desensitization induced by prolonged ACh exposure of the α7 nAChR. Representative current traces of ACh-induced α7 nAChR desensitization by 1 min exposure to 1 mM ACh. Top panel shows the control experiment without additional compound application. The remaining panels show co-application of the indicated compounds with 1 mM ACh (compounds were co-applied 7 s after start of 1 mM ACh application).

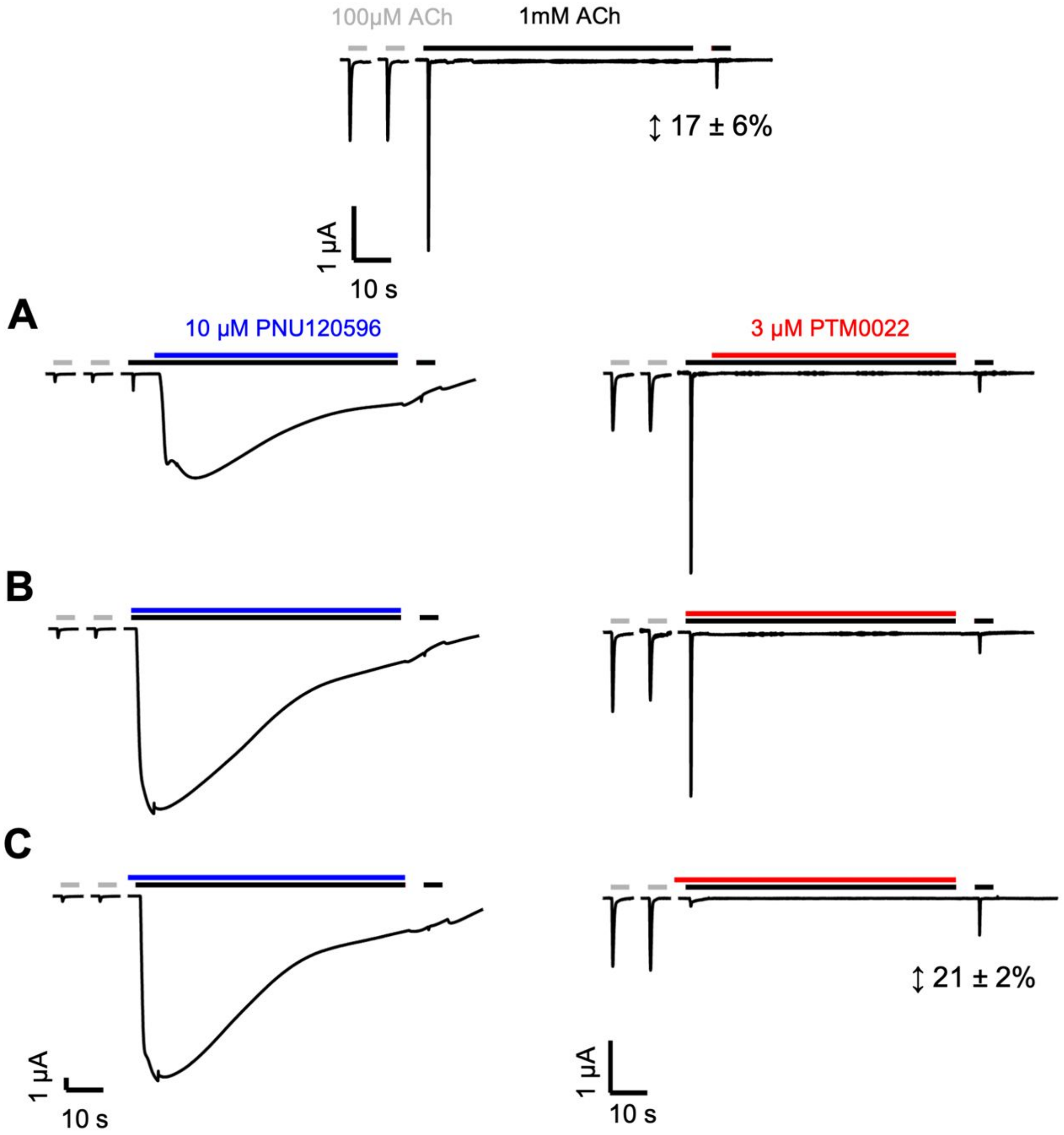

Figure S3: Application of PTM0022 does not recover α7 from desensitization induced by prolonged ACh exposure of the α7 nAChR regardless of time point of application. Representative current traces of ACh-induced desensitization (1 min exposure to 1 mM ACh) of the α7 nAChR. The top panel shows the control experiment without co-application of any compound. The lower three panels show co-/pre-application of 10 µM PNU120596 (left) or 3µM PTM0022 (right) at the following time points (**A**) 7 s after start of ACh-application, (**B**) together with ACh application, (**C**) after 20 s of pre-incubation and during ACh application. Note that traces in panel (A) were already shown in Figure S2. Experiments shown in A), B) and C) were performed subsequentially on one *X. laevis* oocyte for each compound. In case of PNU120596, the next experiment was only performed after washout of PNU120596 (when ACh-evoked responses showed fast desensitization and no sign of modulation). Representative recordings from a three different oocytes per compound are shown. Values in the control experiment and Figure C represent the fraction of the recovery peak (AUC) compared to the last control response to 100 µM ACh (mean ± SD).

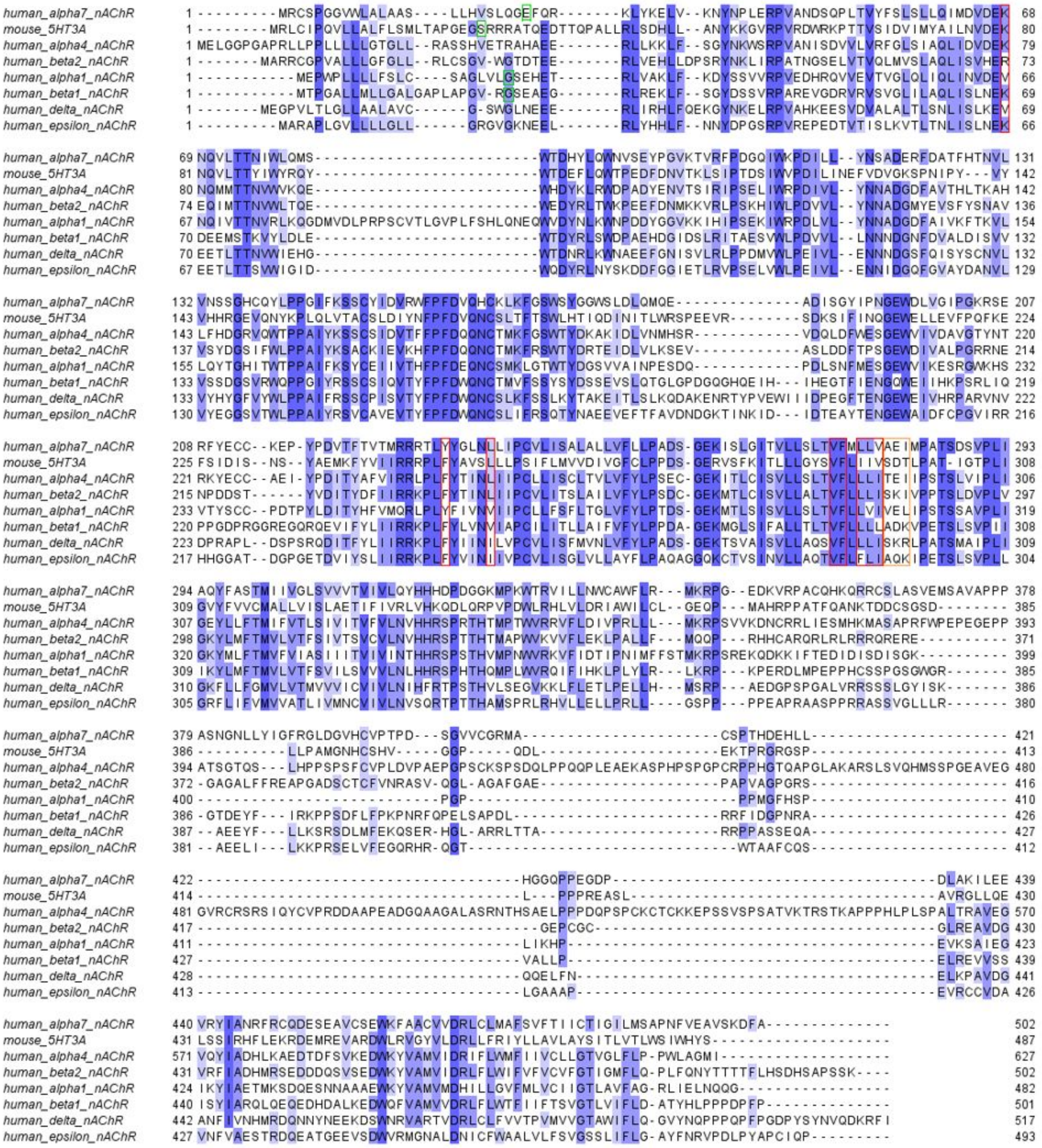

Figure S4: Multiple sequence alignment of cys-loop receptors relevant for this study. Coloring by "percentage identity" with consensus sequence of the alignment, higher identity is indicated by darker blue. Important residues for PTM0022 binding at the α7 nAChR, identified by molecular docking studies, are framed in red, critical residues identified in this study are framed in orange. If residues are counted in one Cys-loop receptor, position 1 is framed in green. Alignment was performed using the "muscle" algorithm in Jalview.

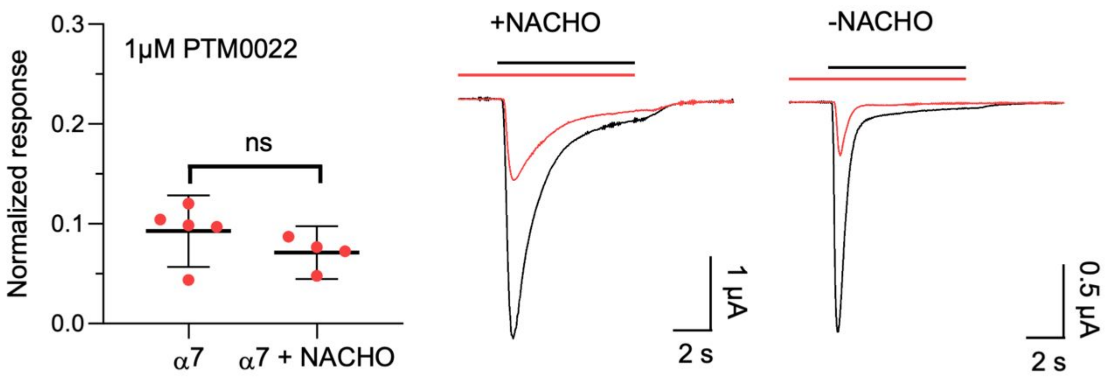

Figure S5: Co-injection of α7 with NACHO does not alter compound effects. Since the α7 SDT mutant required co-injection of NACHO cRNA to form functional receptors, we used wt α7 to test if co-expression of NACHO alters the effect of the compounds as shown here for 1 µM PTM0022 (red line in current traces). Current responses were evoked with 100 µM ACh (black line above current traces). Single values with mean as black bar and S.D. are shown. Note that co-expression of NACHO potentiates current responses. In order to work with comparable currents, α7 alone was injected with 5 ng RNA per oocyte and measured after 2 days and α7 + NACHO was injected with 2.5 ng α7 + 2.5 ng NACHO per oocyte and measured after 1 day.

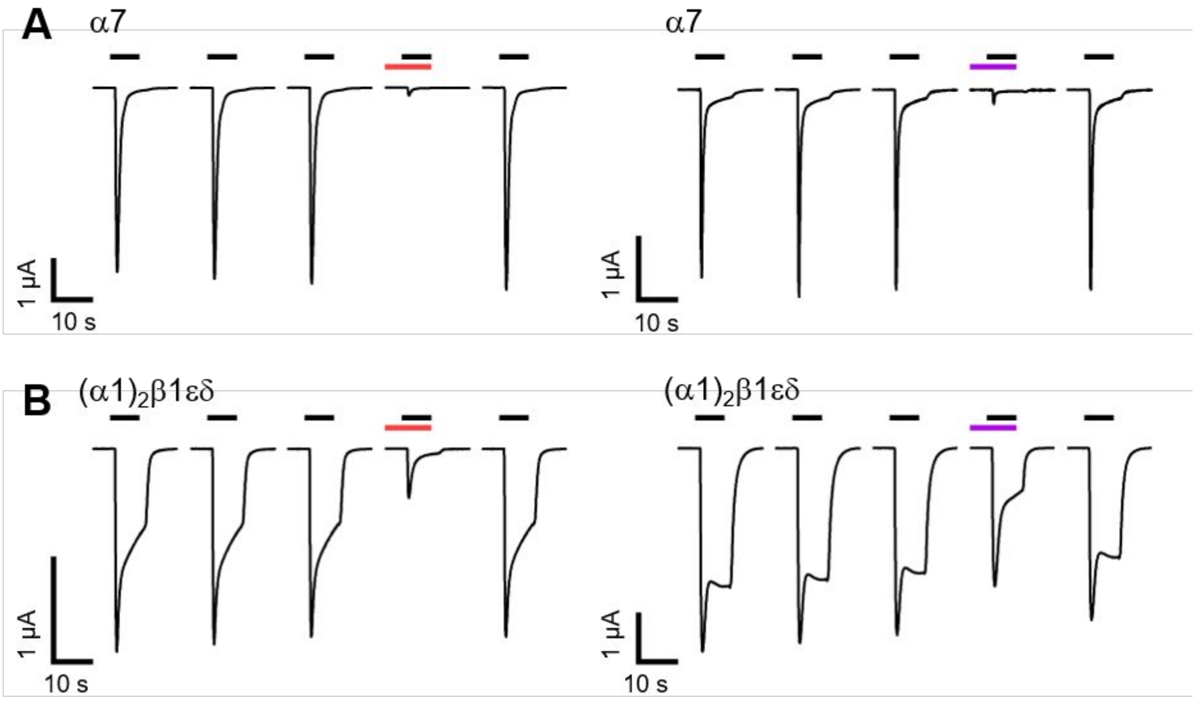

Figure S6: Fast recovery of ACh-induced responses after wash-out of BPCs. Typical current traces of the α7 (**A**) and $(\alpha1)_2\beta1\varepsilon\delta$) muscle-type (**B**) nAChRs elicited by 100 μM and 30 μM ACh, respectively. Three control responses followed by a response after 20 s pre-incubation and co-application of 1 μM PTM0022 (red) or 10 μM MB327 (magenta) and an additional control response are shown. ACh (indicated by bars on top of the current traces) was applied in 2 min intervals (with continuous perfusion of buffer).

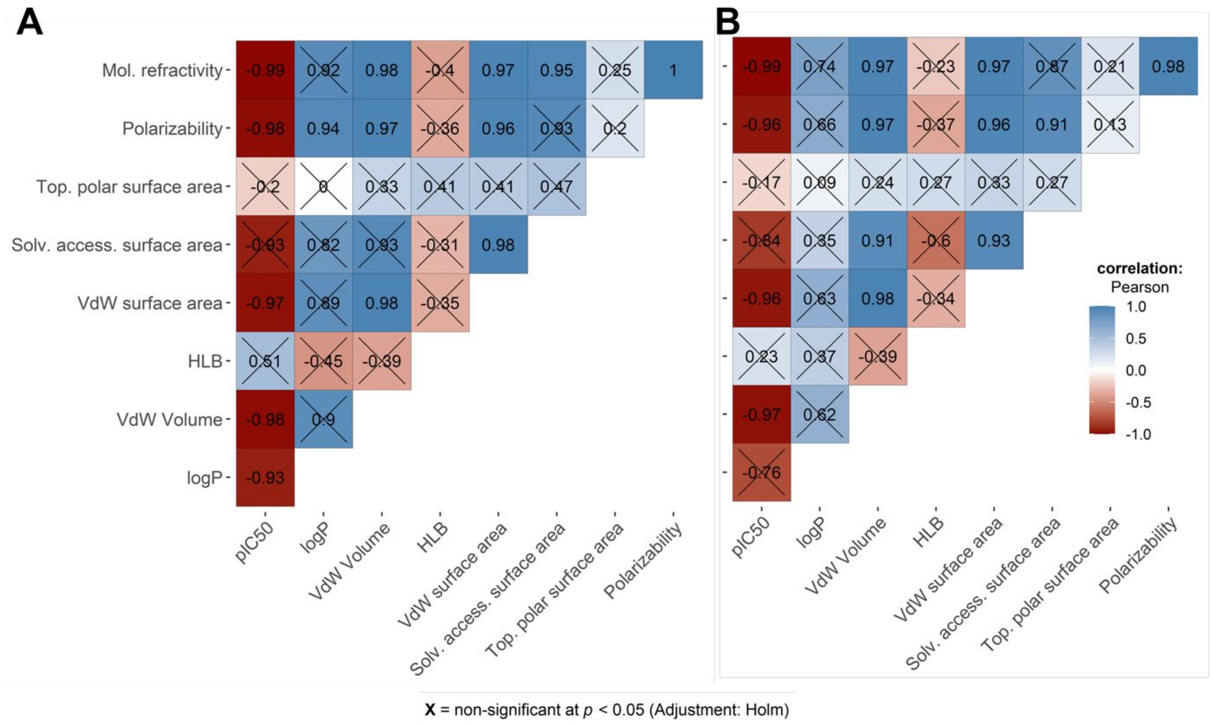

Figure S7: Correlation analysis of the potency of the bispyridinium compounds at the human α7 nAChR and their chemical properties. Pearson correlation of the determined pIC_{50} values and relevant QSAR chemical properties (see supplementary table S5) obtained from chemicalize.com (ChemAxon) with significance threshold of $p < 0.05$ (Holm corrected) without (**A**) ($n = 8$) or with (**B**) ($n = 7$) QX-314. Negative and positive correlations are indicated with red and blue, respectively.

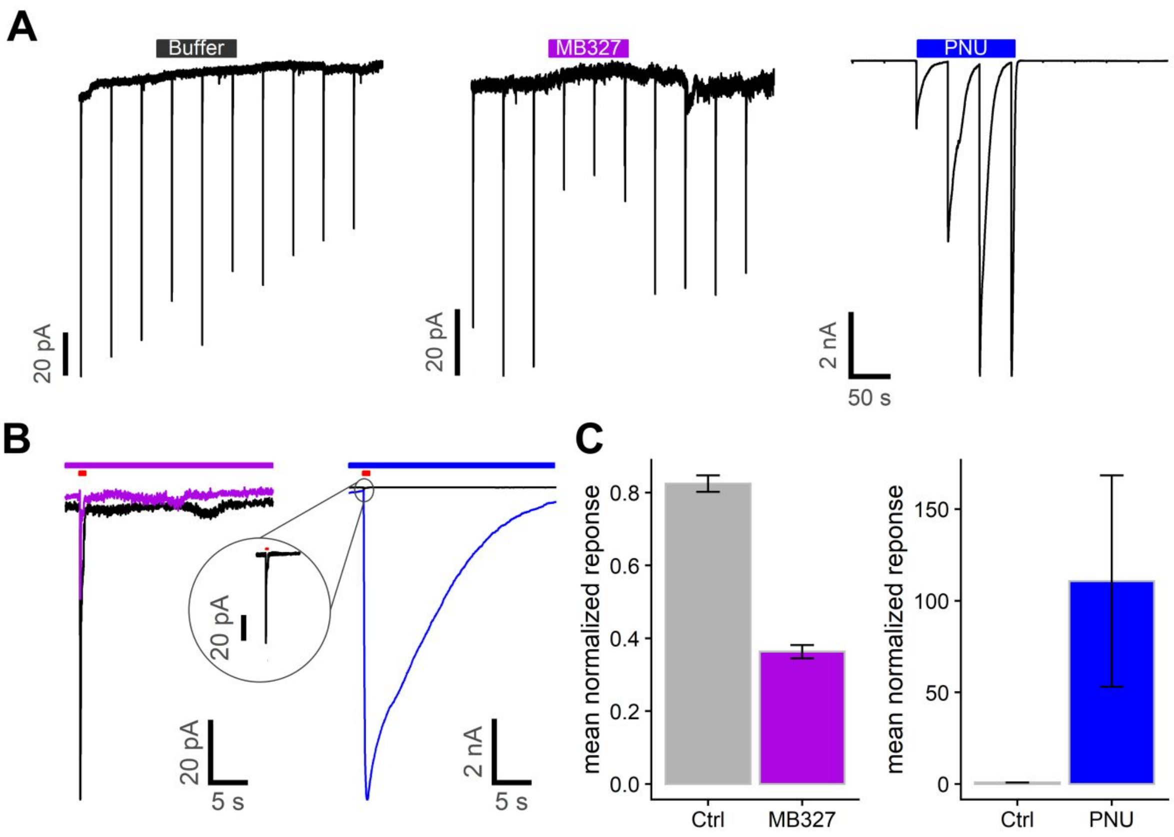

Figure S8: MB327 inhibits human α7 nAChR in stably transfected CHO cells.

(**A**) Manual patch-clamp recordings of inward current responses from human α7 nAChRs evoked by the repeated application (0.5 s) of ACh (100 µM) in the absence (negative control recording; left) or the presence of MB327 (30 µM; middle) or PNU120596 (PNU; 10 µM; right). The first three ACh applications (sweeps 1-3) were used as the control phase, followed by the compound application phase (sweep 4-6) and the washout phase (sweep 7-10). Note the large current increase and altered Y-axis during PNU application. Note that during sweep 3 of the recordings with PNU120596, two current responses were evoked. The first response was triggered by ACh and similar to the two previous ACh-triggered responses (sweeps 1 and 2). The second response was much larger and evoked by the application of PNU120596 during the washout phase.

(**B**) Overlay of representative ACh (100 µM) -evoked current traces of human α7 nAChRs in the absence (control phase; sweep 2; black) and the presence (compound phase; sweep 5) of MB327 (30 µM; left; purple) or PNU120596 (10 µM; right; blue). The inset on the right side displays the enlarged response in the absence of PNU120596 (control phase; sweep 2; black). The traces from the control phase and the compound phase originate from the same cell, respectively. Note the different y-axes. (**C**) Ratio of the peak current responses of human α7 nAChRs triggered by the second and the fifth application (sweep 2 and 5) of ACh (100 µM) during negative control recordings (n = 5) and in the presence of MB327 (30 µM; n = 4) or PNU120596 (PNU; 10 µM; n = 3). The data are depicted as mean ± SEM.

Manual patch-clamp recordings - Methods

Patch-clamp experiments were performed in the whole-cell mode (Hamill *et al.* 1981) using an EPC 10 USB patch-clamp amplifier and the PATCHMASTER software (version 2x91; HEKA Elektronik, Germany). The extracellular solution contained (in mM): 140 NaCl, 5 KCl, 1 $MgCl_2$, 0.5 $CaCl_2$, 10 HEPES and 10 D-glucose, pH 7.4. The intracellular solution contained (in mM): 120 KCl, 2 $MgCl_2$, 10 HEPES, 10 EGTA, 1 sucrose and 31.25 KOH, pH 7.2. All substances were obtained from Carl Roth except EGTA, which was from Sigma Aldrich.
The patch-clamp experiments with CHO cells stably expressing human α7 nAChRs were executed using the Dynaflow Resolve System (Fluicell, Sweden), which allows a fast buffer exchange. The recordings were performed at room temperature and the cells were held at -60 mV in the voltage-clamp mode.
The following application protocol was used for each of the 10 sweeps of an experiment: After a baseline recording for 2 s, ACh (100 µM) was applied for 0.5 s, followed by a washout for 10 s. The first three sweeps were used as a control (control phase). During the third sweep, the application of ACh (100 µM) was followed by the washout of ACh and the simultaneous treatment of the cells with MB327 (30 µM) or PNU120596 (10 µM) for 10 s. After the control phase, the next three sweeps (number four to six) were used for the compound tests (compound phase), where all steps of the application protocol were performed in the presence of MB327 (30 µM) or PNU120596 (10 µM). During the sixth sweep, the application of ACh (100 µM) was followed by a washout for 10 s, without MB327 (30 µM) or PNU120596 (10 µM) present. The compound phase was followed by four additional sweeps (washout phase), which were performed like the first three sweeps (control phase). The negative control recordings were executed as described above but without MB327 (30 µM) or PNU120596 (10 µM) present. Acetylcholine chloride and PNU120596 were obtained from Sigma Aldrich.
The data of the manual patch-clamp recordings were analyzed and visualized with scripts written in R (R Project for Statistical Computing, RRID:SCR_001905) using the following packages: tidyverse (RRID:SCR_019186) and ggplot2 (RRID:SCR_014601).

The current responses were determined as peak currents.
